# How the Arrangement of Platinum Atoms on Ruthenium Nanoparticles Improves Hydrogen Evolution Activity

**DOI:** 10.1002/adma.202509610

**Published:** 2025-07-22

**Authors:** Qinyu Li, Soshan Cheong, Agus R. Poerwoprajitno, Shuting Xiang, Anatoly I. Frenkel, Yuwei Yang, Nicholas M. Bedford, Sohaib Umer, Martina Lessio, Ichiro Ohnishi, Zeno R. Ramadhan, Dale L. Huber, Liming Dai, Wolfgang Schuhmann, J. Justin Gooding, Richard D. Tilley

**Affiliations:** ^1^ School of Chemistry University of New South Wales Sydney NSW 2052 Australia; ^2^ Mark Wainwright Analytical Centre University of New South Wales Sydney NSW 2052 Australia; ^3^ Center for Integrated Nanotechnologies Sandia National Laboratories Albuquerque NM 87185 USA; ^4^ Department of Materials Science and Chemical Engineering Stony Brook University Stony Brook NY 11794 USA; ^5^ Division of Chemistry Brookhaven National Laboratory Upton NY 11973 USA; ^6^ School of Chemical Engineering University of New South Wales Sydney NSW 2052 Australia; ^7^ JEOL Ltd. 3‐1‐2 Musashino, Akishima Tokyo 196‐8558 Japan; ^8^ Analytical Chemistry – Center for Electrochemical Sciences (CES) Faculty of Chemistry and Biochemistry Ruhr University Bochum D‐44780 Bochum Germany; ^9^ Australian Centre for NanoMedicine University of New South Wales Sydney NSW 2052 Australia

**Keywords:** atomic clusters, bimetallic synergy, co‐catalysis, hydrogen evolution reaction, platinum‐ruthenium

## Abstract

The platinum‐ruthenium (PtRu) system is highly active for hydrogen evolution reaction (HER) in alkaline media with both Pt and Ru playing active roles in the water dissociation step that generates adsorbed hydrogen atoms. Precise control of the arrangement of Pt atoms on Ru nanoparticles can maximize the Pt‐Ru sites for water dissociation and Pt‐Pt sites for hydrogen production and can considerably improve HER catalytic performance. By directing the growth and distribution of Pt on Ru hourglass nanoparticles, the arrangement of Pt on Ru is controlled into forming Pt islands, small Pt clusters, and strings of a few Pt atoms. Calculations show that the unique atomic string arrangements of Pt on Ru is the thermodynamically favorable configuration. Additionally, these strings have a favorable combination of Pt‐Ru and Pt‐Pt sites, making the Pt‐string on Ru the most active catalyst with a more than fivefold increase in turnover frequency for alkaline HER compared to the Pt‐island on Ru catalyst. The results show how controlling the Pt atomic arrangement on Ru nanoparticle surfaces for the tuning of Pt‐Pt and Pt‐Ru neighboring sites can direct toward a more efficient HER mechanism and thereby significantly enhancing HER performance.

## Graphical abstract

A synthetic strategy for the formation of Pt islands and their transformation into Pt atomic strings on Ru nanoparticle surfaces is presented. The thermodynamically favored configuration of Pt strings on Ru catalyst is demonstrated to catalyze HER via the faster Volmer–Tafel pathway with a significantly improved intrinsic activity, owing to the well‐aligned Pt‐Ru sites adjacent to Pt‐Pt sites.

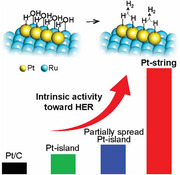

## 1 Introduction

Pt‐Ru is an excellent bimetallic system for accelerating both the water dissociation and the hydrogen formation steps in the electrocatalytic hydrogen evolution reaction (HER) in alkaline electrolytes. This is because the oxophilic Ru enhances the first step by lowering the energy for water adsorption and dissociation to generate adsorbed hydrogen atoms (H_ads_) on the Pt catalyst active site,^[^ ^1^ ^]^ whereas the H‐binding energy of Pt makes it the most active metal for the second step in the HER process, the formation of a hydrogen molecule and its desorption from the catalyst.^[^ ^2^ ^]^ We can envision that an ideal active site for HER will consist of a configuration where there are multiple Ru‐Pt pairs to enhance the water dissociation step, which then have adjacent Pt‐Pt sites able to accept 2 H_ads_ to be combined to form and desorb H_2_ by the Tafel step. Thus, controlling the atomic configuration of Pt on Ru is vital for enhancing catalytic performance.

An effective approach would be to strategically decorate Pt atoms on Ru nanoparticle surfaces. Having Pt on the surface rather than embedded in the particle also ensures efficient utilization of the more expensive Pt. Controlling Pt decoration on Ru nanomaterial surface is highly challenging, even more so for constructing Pt‐Ru and Pt‐Pt neighboring sites for catalysis. In nanoparticle synthesis, co‐reduction of Ru and Pt precursors typically yields alloys of Pt‐Ru having a random arrangement of the two metals,^[^ ^3^ ^]^ whereas growing or depositing Pt on Ru usually results in Ru‐core Pt‐shell structures.^[^ ^4^ ^]^ Our group has successfully developed a synthetic strategy where mild reduction conditions allow the slow growth of small, few‐nm Pt islands on metal nanoparticles.^[^ ^5^ ^]^ We have also developed a methodology to spread the Pt islands to form single‐atom Pt catalysts.^[^ ^5a^ ^]^ When these two synthetic approaches are combined, they offer an opportunity to control the arrangement and distribution of Pt‐Ru and Pt‐Pt neighboring sites on the nanoparticle surfaces.

In this study, Pt‐Ru and Pt‐Pt active sites are formed by strategically growing and then spreading Pt islands on Ru hourglass‐shaped nanoparticles. As the islands spread, the Pt atoms form into atomic strings and clusters dispersed on the Ru surfaces. These structures are shown by calculations to be the thermodynamically favorable configurations. The Pt string decorated Ru nanoparticles exhibit excellent catalytic activity with a low overpotential and a high turnover frequency being 5.4 and 3.5 times more active than the Pt islands and partially spread islands on Ru nanoparticles, respectively. The intrinsic activity improvement of over 9 times more active than a commercial Pt/C catalyst, is among the highest reported for Pt‐Ru catalyst systems. This enhancement is due to the co‐existence of both Ru‐Pt and Pt‐Pt sites, forming active sites with the desired configuration for accelerating both water dissociation and hydrogen formation steps in alkaline HER.

## 2 Results and Discussion

**Figure 1:**
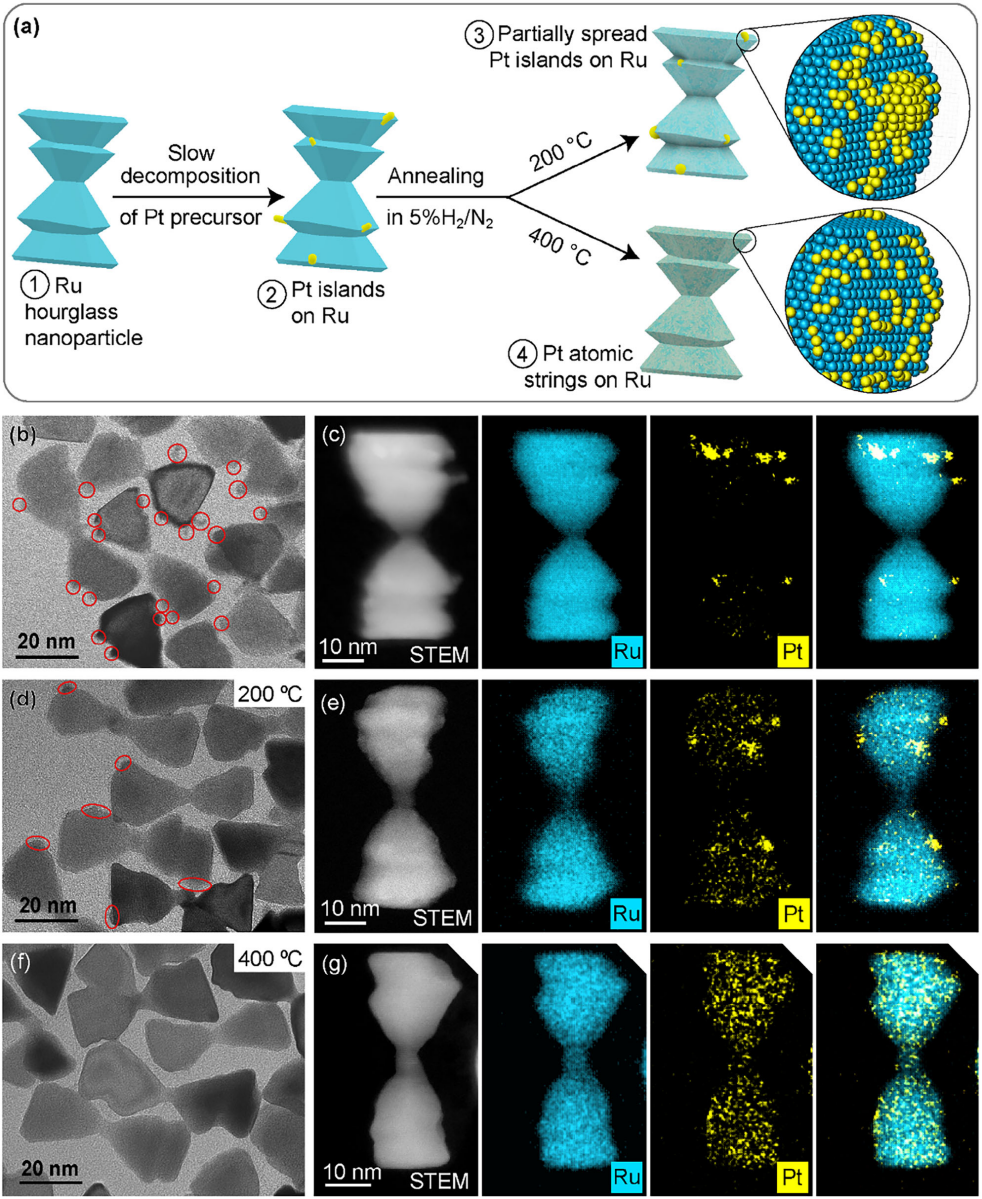
a) Schematic illustration of the preparation of hourglass‐shaped Ru nanoparticles decorated with Pt islands, partially spread islands, and Pt atomic strings. b) TEM image of Ru nanoparticles decorated with Pt islands, with red circles marking the islands on the step‐edges and corners of the hourglass branches. c) STEM image and EDX elemental maps of a Pt‐island on Ru nanoparticle. d) TEM image of nanoparticles after annealing at 200 °C, with red ovals marking the partially spread Pt islands. e) STEM image and EDX elemental maps of a Ru nanoparticle decorated with partially spread Pt islands. f) TEM image and g) STEM image and EDX elemental maps of fully spread Pt on Ru nanoparticles obtained from 400 °C annealing.

The controlled decoration of Pt on Ru nanoparticles was achieved by directed growth and spreading of Pt islands on Ru nanoparticles under a controlled environment, as illustrated in the scheme in **Figure** **1**a.

The Ru nanoparticles for Pt decoration were prepared by a thermal‐reduction method,^[^ ^6^ ^]^ in which ruthenium(III) acetylacetonate dissolved in a mixture of 1‐octadecene as solvent and dodecylamine as stabilizer was heated at 140 °C under 1 bar of hydrogen. The use of 1‐octadecene as a non‐coordinating solvent and a relatively low Ru precursor concentration enabled a slow growth over 48 hours to form hourglass‐shaped nanoparticles with multiple step‐edges (Figure S1, Supporting Information), depicted as structure (1) in Figure 1a. The hexagonal close‐packed (hcp) crystal structure of the Ru nanoparticles is confirmed by selected area electron diffraction (SAED, Figure S2, Supporting Information).

The hourglass morphology of the Ru nanoparticles was chosen because the nanoparticle surfaces are bound by the low‐index {0001} and {10$\bar{1}$1} facets (Figure S3, Supporting Information). These low‐index facets are known to offer high catalytic stability due to the relatively high coordination numbers of the surface atoms,^[^ ^7^ ^]^ making them highly suitable as a substrate material for designing catalytic active sites. Another feature of the shape of the Ru nanoparticles are the multiple step‐edges, which are the exposed sites for Pt nucleation and growth.^[^ ^8^ ^]^

The Pt islands were grown onto the Ru nanoparticles following a slow injection method with the Pt precursor solution being introduced at 0.2 mL min^−1^ to the Ru nanoparticle suspension. The slow injection prevents homogeneous nucleation of Pt and facilitates preferential nucleation of Pt on the step‐edges and corners of the Ru nanoparticles, as depicted in Figure 1a, structure (2). Annealing the nanoparticles under flowing hydrogen (5% H_2_/N_2_) caused the Pt to spread across the Ru nanoparticle surface. The extent to which the Pt spreads is controlled by the annealing temperature, with flattened and smaller islands or clusters formed at 200 °C, and Pt atoms in string‐like arrangements at 400 °C, which are depicted as structures (3) and (4) in Figure 1a, respectively.

A transmission electron microscopy (TEM) image of the Pt islands on Ru nanoparticles is shown in Figure 1b, where small islands (marked by red circles) can be observed on the hourglass nanoparticles. Formation of face‐centered cubic (fcc) Pt is evident by the Pt(200) spots at 5.26 nm^−1^ in the SAED pattern (Figure S4, Supporting Information). Scanning‐TEM coupled with energy‐dispersive X‐ray spectroscopy (STEM‐EDX) analysis shows that the Pt islands had preferentially nucleated and grown on the step‐edges and corners of the Ru nanoparticles (Figure 1c). The hourglass branches are 21 ± 4 nm in length and 19 ± 3 nm in width (Figure S5, Supporting Information), similar to those of the bare Ru nanoparticles (Figure S1c,d, Supporting Information); and the islands have a height of 4 ± 2 nm and a width of 2.0 ± 0.5 nm on average (Figure S6, Supporting Information).

After being annealed at 200 °C, the Pt islands were partially spread and displayed a flatter appearance as shown by TEM image in Figure 1d. The spread of Pt is also confirmed by STEM‐EDX mapping (Figure 1e). The Pt islands were fully spread when annealed at 400 °C, where the protruding islands seen before heat treatment are no longer visible in the corresponding TEM images (Figure 1f), and the nanoparticles display similar morphology to those of Ru nanoparticles prior to Pt decoration. STEM‐EDX elemental mapping analysis indicates that Pt is uniformly distributed across the nanoparticles, as shown in Figure 1g and Figure S7 (Supporting Information). Apart from the disappearance of the Pt(200) spots, the SAED pattern indicates no change to the hcp structure of the Ru nanoparticles (Figure S8 and Table S1, Supporting Information), showing the stability of the Ru nanoparticles over the course of annealing.

Spreading of the Pt island was found to be best conducted between 200 and 400 °C. An annealing temperature of 150 °C was found to be too low to initiate noticeable change to the islands. Annealing at higher temperatures, such as 600 °C, caused the disappearance of the step‐edge features and sintering of Ru branches in close proximity (Figure S9, Supporting Information). It was also found to be more effective to control the spreading and form different Pt configurations by temperature than by varying the annealing time. Both Pt‐decorated Ru nanoparticles shown in Figure 1d,f were obtained after 2 h of annealing at the respective temperatures. The partially spread Pt islands were still observed after 10 h at 200 °C, and similarly the nanoparticle morphology for nanoparticles annealed at 400 °C for 5 h remained the same as those shown in Figure 1f (Figure S10, Supporting Information).

From these observations we can understand the growth of the Pt on the Ru substrate nanoparticles. Pt is preferentially nucleating and growing on the most exposed sites being the step‐edges and corners of the Ru nanoparticles that are the most exposed. The preferential nucleation is facilitated by the slow decomposition of the Pt precursor that enables site‐specific nucleation.^[5c,^ ^9^ ^]^ When the nanoparticles are annealed, the Pt spreads across the Ru surfaces including the side {10$\bar{1}$1} and base {0001} facets of the hourglass shape. The dispersion of Pt is facilitated by the mobilization of Pt under mild annealing in a H_2_ atmosphere and driven by the formation of strong Ru‐Pt bonds to give a uniform dispersion of Pt across the nanoparticle surface.^[^ ^5a^ ^]^

**Figure 2:**
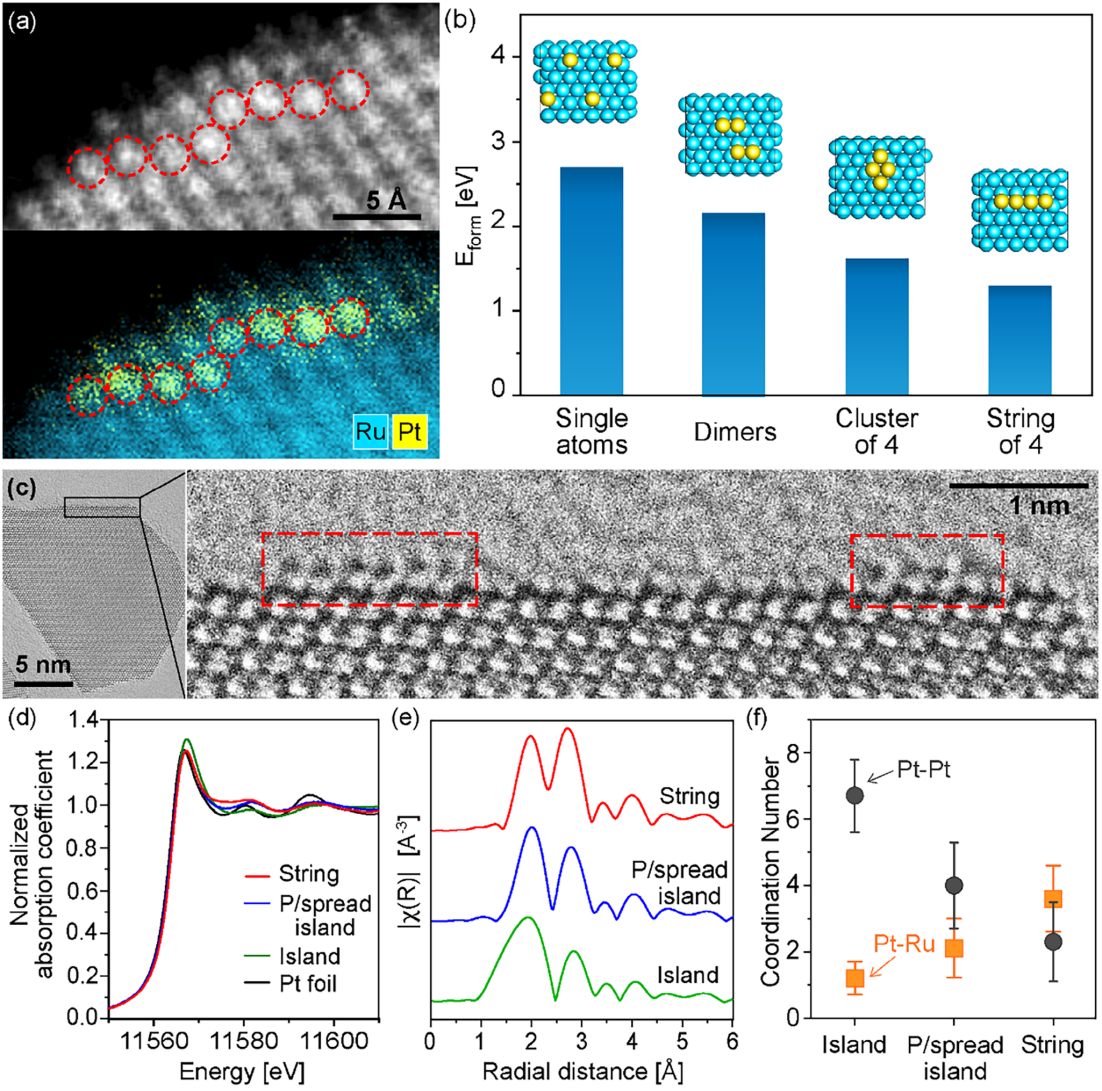
a) Atomic‐resolution STEM image and overlay EDX map of Pt and Ru showing a string‐like pattern of Pt atoms on a Ru nanoparticle surface, with red circles marking the positions of Pt atoms identified by EDX. b) Calculated energy of formation (*E_form_*) for different configurations of Pt atoms on Ru(0001) surface. c) HRTEM image of a branch surface where strings of atoms atop the surface can be observed, as indicated by the red boxes. d) Normalized XANES spectra in reference to a Pt foil at Pt L_3_‐edge. e) R‐space EXAFS spectra of Pt‐string on Ru, partially spread Pt‐island on Ru and Pt‐island on Ru (the k‐range for Fourier transforms was from 2 to 9 Å^−1^ for all spectra), and f) the corresponding EXAFS analysis results for Pt to Pt (Pt‐Pt) and Pt to Ru (Pt‐Ru) coordination numbers.

In high‐resolution high‐angle annular dark‐field (HAADF) STEM imaging, variations of intensity can be observed across the lattice near the edge of the nanoparticles, where the brighter atoms can be interpreted as the presence of Pt (Figure S11, Supporting Information). These brighter atoms are observed to have formed into 3–10‐atom string arrangements, where the Pt atoms are connected in a line; or cluster arrangements, where the Pt atoms are grouped together but not in a line. These atoms make up 66% of the atoms analyzed, while the remainder being single atoms or dimers (Figure S12, Supporting Information). Atomic‐resolution STEM‐EDX mapping verifies that these brighter contrast atoms correlate to the localization of Pt, where atoms are shown to be linked together in a string‐like morphology (**Figure** **2**a; Figure S13, Supporting Information). The STEM contrast difference is most apparent near the edges, implying low concentration of Pt relative to Ru in the lattice.

To gain insights into the stable formations of Pt atoms on Ru surfaces, density functional theory (DFT) was employed to calculate the energy of formation (*E_form_*) of Pt atoms on Ru(0001) surface. Ru(0001) was chosen as a representative surface because the Ru hourglass structures are predominantly composed of low‐index facets such as Ru(0001). The fully spread Pt can form into single atoms, dimers, or groups of a few atoms. For four Pt atoms on Ru(0001), the possible configurations are four isolated single atoms, two pairs of dimers isolated from each other, or atoms connected together in a four‐atom cluster or string‐like arrangements, as depicted in Figure 2b. The calculated *E_form_* values indicate that the arrangement into a string is more stable compared to a cluster or single atom configurations. This can be attributed to a stronger Pt‐Pt interaction in the string configuration, which also allows closer Pt‐Pt distances (Table S2, Supporting Information). The more favorable interaction also means Pt atoms prefer to adsorb in the vicinity of other Pt atoms on the Ru surface rather than scattering, hence strings and clusters are preferred over single atoms and dimers.

Further evidence for strings of atoms on the surface of the Ru nanoparticles is shown in Figure 2c, a TEM image acquired under low‐electron dose conditions with the nanoparticles deposited onto a low‐background graphene support film. The Pt atoms are clearly observed to be on the surface of the nanoparticle, showing that the Pt islands spread into groups of atoms dispersed onto the surface of the Ru nanoparticle.

The Pt‐Ru and Pt‐Pt coordination of the nanoparticles were studied using X‐ray absorption near‐edge structure (XANES) and extended X‐ray absorption fine structure (EXAFS) spectroscopy. Both Pt and Ru were found to be metallic for all the samples as shown by the similarities of the normalized Pt L_3_‐edge and Ru K‐edge XANES spectra with those for Pt and Ru foils, respectively (Figure 2d; Figure S14, Supporting Information). Pt‐Pt and Pt‐Ru first‐shell coordination numbers were obtained from analysis of the data in the ≈1–3 Å region in the Fourier‐transform magnitudes of the EXAFS (FT‐EXAFS) spectra at Pt L_3_‐edge (Figure 2e; Figure S15, Supporting Information). It is revealed that the Pt‐Pt and Pt‐Ru coordination numbers are different for the three samples, as shown in Figure 2f, which were obtained from fitting of the Pt L_3_‐edge EXAFS data (Figures S16–S18 and Table S3, Supporting Information). As can be seen from Figure 2f the Pt‐Ru coordination number increases and the Pt‐Pt coordination number decreases as the samples transition from Pt islands to partially spread islands and to Pt strings, consistent with Pt transitioning from the Pt‐rich regions to the Ru surface, decreasing the number of Pt‐Pt bonds and increasing the number of Pt‐Ru bonds. Additionally, X‐ray photoelectron spectroscopy (XPS) analysis has been performed on the Pt‐island and Pt‐string on Ru nanoparticles (Figure S19, Supporting Information), which further supports the HAADF‐STEM and EXAFS results that the Pt atoms are mostly in small, atom‐thick cluster or string configurations.

The new approach presented here enables the construction of co‐existing Pt‐Ru and Pt‐Pt active sites with an unprecedented level of control, which has not been previously achieved. The key to this precise arrangement of the active sites is a combination of uniform Pt decoration and controlled Pt spreading on faceted Ru nanoparticles. The low‐index facets of Ru also offer unique sites that not only support Pt decoration but also contribute to the enhanced stability of the active sites.

**Figure 3:**
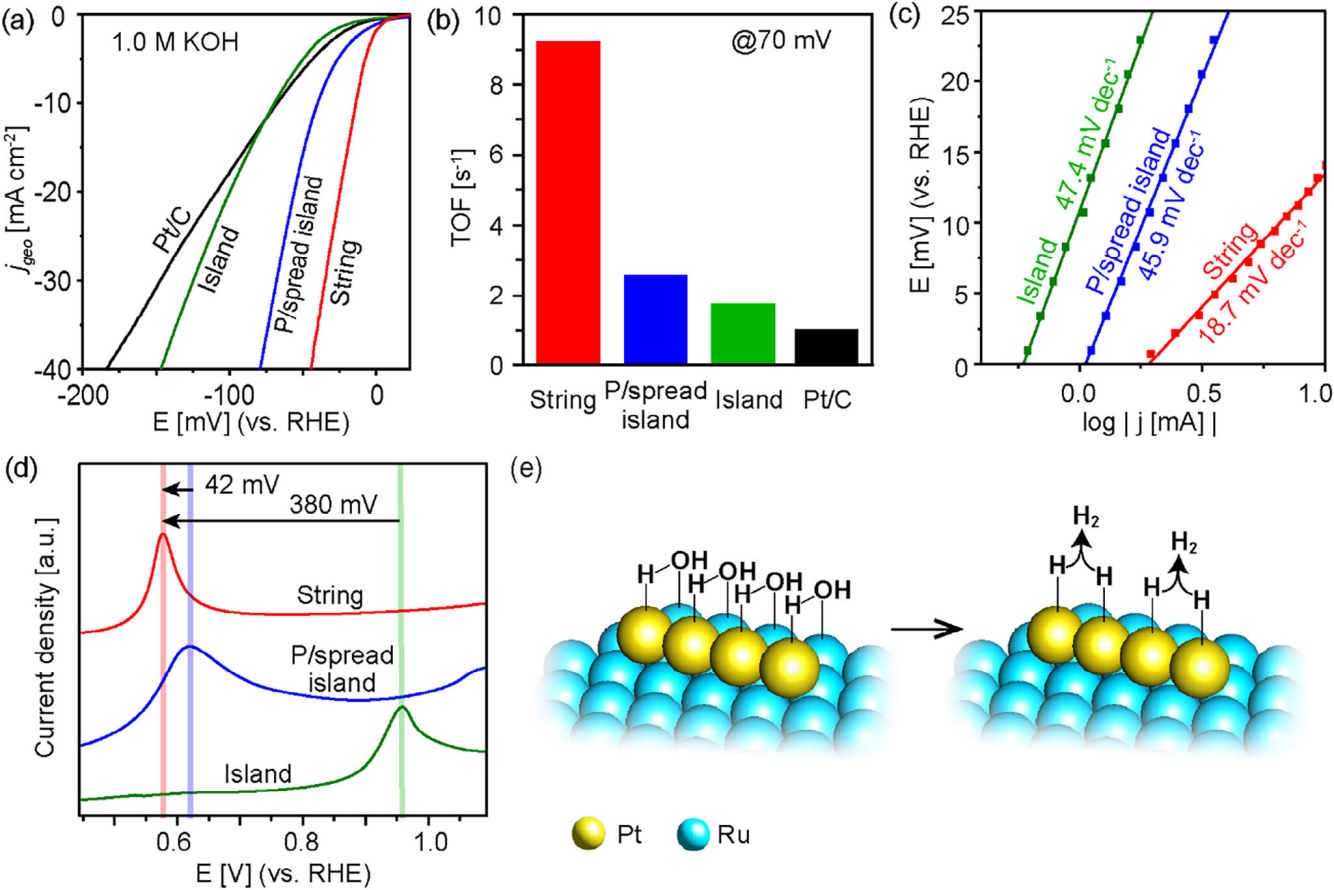
a) HER polarization curves of Pt‐string, partially spread (p/spread) Pt‐island and Pt‐island decorated Ru nanoparticle catalysts and a commercial Pt/C catalyst. b) TOF comparison at 70 mV overpotential. c) Tafel plots of Pt‐string, partially spread Pt‐island and Pt‐island on Ru derived from the respective polarization curves. d) A zoomed‐in region of the CVs at the potential window capturing CO oxidation peak of Pt string, partially spread Pt‐island, and Pt‐island on Ru catalysts. e) Scheme of proposed HER process on the Pt‐string on Ru catalyst.

The HER performance of the Pt‐decorated Ru nanoparticles supported on carbon was studied using a standard three‐electrode system in N_2_‐saturated 1.0 м KOH electrolyte. The Pt‐string on Ru nanoparticle catalyst is the most active among the catalysts tested, as shown in the linear sweep voltammetry (LSV) curves in **Figure** **3**a. To achieve a current density of 10 mA cm^−2^, the Pt‐string on Ru catalyst requires a much lower overpotential of 14 mV, compared to the partially spread (p/spread) Pt‐island on Ru (39 mV), Pt‐island on Ru (69 mV), and Pt/C (67 mV) catalysts. While the Pt‐island catalyst exhibits similar activity to that of the commercial Pt/C, it outperforms the Ru hourglass nanoparticles with no Pt decoration, which requires an overpotential of 177 mV to achieve 10 mA cm^−2^ (Figure S20, Supporting Information). This shows that the primary active sites of the Pt‐decorated Ru catalysts are those that contain Pt.

The dispersion of Pt on the Ru nanoparticles results in at least an order of magnitude higher activity per mass of Pt (86, 28, 10 A mg^−1^_Pt_ for Pt‐string, partially spread Pt‐island, and Pt‐island catalysts, respectively) compared to Pt/C (0.18 A mg^−1^_Pt_, Figure S21a, Supporting Information). When considering the mass of both Pt and Ru metals, the Pt‐string on Ru catalyst (0.56 A mg^−1^) is 3 times and >9 times more active than the partially spread Pt‐island (0.18 A mg^−1^) and Pt‐island (0.06 A mg^−1^) catalysts, respectively (Figure S21b, Supporting Information). The enhanced performance of the Pt‐string on Ru catalyst is also demonstrated by its high turnover frequency (TOF) of 9.2 s^−1^ at 70 mV vs. RHE, representing HER efficiency that is 3.5 times higher than the partially spread Pt‐island (2.6 s^−1^), 5.4 times higher than the Pt‐island (1.7 s^−1^), and 9.2 times higher than the Pt/C (1.0 s^−1^), as shown in Figure 3b. At 100 mV vs RHE, the TOF value of 15.3 s^−1^ obtained for the Pt‐string on Ru catalyst is among the highest reported for Pt‐Ru catalysts for HER in alkali (Table S4, Supporting Information).

The HER reaction typically proceeds in a two‐step process that may follow two mechanisms. The Volmer step involves the dissociation of water and adsorption of H from water, and is the initial step in the HER reaction. From here the adsorbed H (H_ads_) may follow the Heyrovsky mechanism where it reacts with a proton and an electron yielding molecular hydrogen (H_2_) that is then released from the surface. Alternatively, the H_ads_ may dimerize with a second H_ads_ to yield H_2_, in the Tafel mechanism. The Volmer–Tafel mechanism is faster and more efficient than the Volmer–Heyrovsky mechanism, but to proceed quickly it requires that multiple H_ads_ be generated in close proximity. So, an ideal catalyst would have multiple Pt‐Ru sites to generate H_ads_, where the oxophilic Ru facilitates the dissociation of water for the Volmer step. This ideal catalyst would also have nearby Pt‐Pt sites to catalyze the Tafel mechanism.

It appears that the Pt‐string on Ru morphology has a nearly ideal arrangement of Pt‐Ru and Pt‐Pt sites to allow the Volmer–Tafel mechanism to proceed efficiently. This efficiency is reflected by its low Tafel slope (18.7 mV dec^−1^) that corresponds to the Volmer–Tafel pathway for alkaline HER (Figure 3c). The desirable placement of Pt and Ru sites is also implied when probed with a complementary CO stripping reaction (Figure 3d; Figure S22, Supporting Information). Compared to HER, the CO stripping reaction also begins with a Volmer step producing H_ads_ and OH, but utilizes OH to oxidize an adsorbed CO molecule.^[^ ^10^ ^]^ In the presence of an oxophilic metal next to Pt, the CO adsorbed on Pt reacts with the OH on the oxophilic metal at a lower or more negative potential as a result of more readily available OH species.^[^ ^11^ ^]^ For the Pt‐string on Ru catalyst, the ‐380 mV shift in the CO oxidation potential relative to that of the Pt‐island catalyst clearly suggests the close proximity of the Pt atoms to Ru atoms in the Pt‐string catalyst.

In contrast, both the Pt‐island and partially spread Pt‐island catalysts have similar Tafel slopes, 47.4 and 45.9 mV dec^−1^, respectively, which are similar to those obtained for the Ru hourglass nanoparticles and the commercial Pt/C catalyst (Figure S23, Supporting Information). These Tafel slopes suggest a Volmer–Heyrovsky pathway, due to the low number of Pt‐Ru sites leading to a low concentration of H_ads_ from the Volmer step. Although the partially spread Pt‐island catalyst exhibits an enhanced Volmer step (relatively negative CO oxidation peak, Figure 3d), the Pt‐Ru active sites are much less uniform as indicated by the broad CO oxidation peak (0.5–0.8 V), and HER is still limited by the lack of adjacent H_ads_ as a prerequisite for the Tafel mechanism to take place. In comparison, for the Pt‐string catalyst, all Pt‐Pt sites are adjacent to Pt‐Ru sites, so each H_ads_ generated during the Volmer step is directly located next to another H_ads_ for an efficient H_2_ evolution via the Tafel mechanism (Figure 3e).

**Figure 4:**
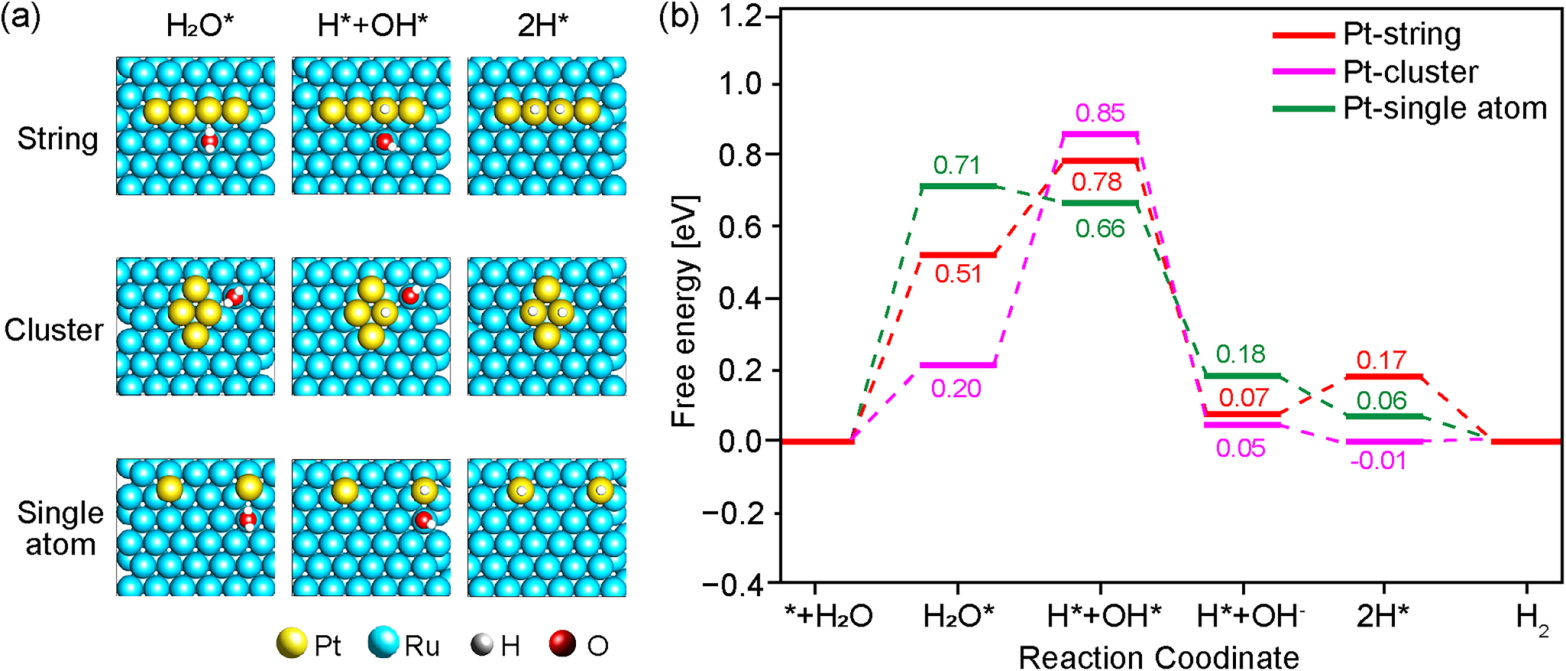
a) Optimized geometries of key HER intermediates adsorbed on the three distinct Pt configurations: Pt‐string, Pt‐cluster, and Pt‐single atom on a Ru(0001) surface. b) Gibbs free energy profiles for alkaline HER over the three Pt configurations.

To gain understanding of the HER mechanism for the Pt‐string on Ru catalyst, the Gibbs free energy profile was calculated. The Pt configurations considered were the string, cluster and single‐atom on Ru(0001) determined from the energy of formation (*E_form_*) calculation (Figure 2b). The Pt‐string and Pt‐cluster configurations have four Pt atoms adsorbed on the Ru(0001) surface, whereas the Pt‐single atom configuration was modelled by placing two Pt atoms far from each other. The optimized geometries of key HER intermediates adsorbed on Pt‐string, Pt‐cluster, and Pt‐single atom configurations are shown in **Figures** **4**a and S24 (Supporting Information).

The Gibbs free energy profiles in Figure 4b reveal that the Pt‐string configuration exhibits a moderate energy barrier (Δ*G*) of 0.51 eV for water adsorption, indicating an intermediate binding strength. In comparison, Pt‐cluster demonstrates a stronger interaction with water having a Δ*G* of 0.20 eV, whereas Pt‐single atom configuration shows a significantly weaker interaction with a Δ*G* of 0.71 eV. In the subsequent dissociation of water into *H and *OH, Pt‐string exhibits an increase in the free energy value of 0.27 eV, which is much lower than the dissociation of water on the Pt‐cluster configuration with a Δ*G* of 0.65 eV. This means that the Pt string configuration has a perfect balance between water adsorption and water dissociation. The *H adsorption is thermodynamically favorable over all the Pt configurations. However, an optimal binding energy (not too strong nor too weak) of two hydrogen atoms on the Pt‐string configuration facilitates the recombination and desorption of H_2_ from the catalyst surface. The DFT findings confirm that Pt‐strings adsorbed on a Ru surface are favored for the Volmer–Tafel pathway.

In terms of stability, under constant current density of 10 mA cm^−2^ in 1 м KOH, the Pt‐string on Ru catalysts remained active for over 20 h with a small overpotential increase of 10.7 mV (Figure S25, Supporting Information). The Pt‐island on Ru catalyst was also stable with an overpotential increase of 34.8 mV after 20 h. In contrast, the Pt/C catalyst had a >140 mV increase in overpotential after only 10 h. Additionally, after 10 h operation at a higher current density of 100 mA cm^−2^, an overpotential increase of 62 mV for the Pt‐string on Ru catalyst was observed showing good relative stability at higher currents (Figure S26, Supporting Information). The high stability can be ascribed to the strong Pt‐Ru bond that prevents Pt dissolution and aggregation, as well as to the hcp Ru support with well‐defined morphology that is known to withstand harsh alkaline HER conditions.^[^ ^12^ ^]^ TEM imaging, SAED and STEM‐EDX analysis of the Pt‐string on Ru catalyst after stability test at 10 mA cm^−2^ in 1 м KOH confirm the structural stability of Ru, that the nanoparticle shape is retained and that Pt remains uniformly distributed over the Ru nanoparticles (Figure S27, Supporting Information).

## 3 Conclusion

We presented a synthetic strategy for decorating Pt on Ru nanoparticles to construct co‐existing Pt‐Ru and Pt‐Pt active sites for enhancing electrocatalytic HER in alkaline media. The active sites being derived from unique string‐like arrangements of Pt atoms distributed on Ru nanoparticle surfaces were achieved by directed growth and controlled spreading of Pt islands on hourglass‐shaped Ru nanoparticles. The unprecedented level of synthetic control has enabled HER performance of different Pt configurations on Ru nanoparticles to be investigated. The well‐aligned Pt‐Pt sites adjacent to Pt‐Ru sites catalyze alkaline HER via the more efficient Volmer–Tafel instead of the Volmer–Heyrovsky pathway typically followed by Pt‐Ru catalysts. This work provides new perspectives on how precise control of atomic arrangement of Pt on Ru improves the intrinsic catalytic properties of Pt‐Ru systems and illustrates the opportunity offered by different arrangements of Pt for enhancing catalytic properties. This precise control of atomic arrangement will drive catalyst design for reactions involving complementary or co‐catalytic active sites.

## 4 Experimental Section

### Materials

Ruthenium(III) acetylacetonate (Ru(acac)_3_, 97%), platinum(II) acetylacetonate (Pt(acac)_2_, 97%), dodecylamine (DDA, 98%), oleylamine (OLA, 70%), 1‐octadecene (ODE, 90%), benzyl ether (98%) and Nafion (Nafion 117) were purchased from Sigma Aldrich. Toluene (99.6%) was purchased from Chem‐Supply. Hexane (99%) was purchased from SupraSolv. Carbon black (Vulcan XC 72R) was purchased from Fuel Cell Store. Isopropanol (propan‐2‐ol, 99.5%) was purchased from Chem‐Supply.

### Synthesis of Hourglass‐Shaped Ru Nanoparticles

A mixture of 0.1 mmol Ru(acac)_3_, 0.5 mmol DDA, and 5 mL ODE was sonicated for 5 min, and transferred to a 10‐mL vial. In a typical synthesis, 4 vials of precursor mixture were prepared and placed in a 100‐mL Fisher‐Porter reaction vessel, which was then evacuated and filled with 1 bar of H_2_ and reacted in an oven at 140 °C for 48 h. The reaction vessel was allowed to cool to room temperature before being opened to air. The nanoparticles were purified and collected by repeated centrifugation (1500 rcf, 5 min) of dispersion in 8 mL toluene. The collected precipitate was dried in flowing N_2_ to remove residual solvent and the purified Ru nanoparticles were stored as a powder in a glove box until further use.

### Decoration of Pt on Ru Nanoparticles

Growth of Pt islands on Ru nanoparticles was performed in a three‐necked round‐bottom flask attached to a Schlenk line setup. 0.16 mmol Ru nanoparticles suspended in 5 mL ODE were heated to and maintained at 180 °C under magnetic stirring for 30 min under flowing Ar. 0.036 mmol Pt(acac)_2_ in a mixture of 1.4 mmol OLA and 1 mL benzyl ether was injected into the Ru nanoparticle suspension using a syringe pump at 0.2 mL min^−1^. The reaction was kept at 180 °C for 20 min, and was then allowed to cool to room temperature under an Ar atmosphere. The Pt‐island decorated Ru nanoparticles were purified by repeated centrifugation (1500 rcf, 5 min) of dispersion in toluene and dried under flowing N_2_. To spread the Pt islands and prepare Pt‐string decorated Ru nanoparticles, the Pt‐island on Ru nanoparticles were first loaded onto carbon followed by annealing in a tube furnace. 5 mg of Pt‐island on Ru nanoparticles and 16 mg of carbon black in 5 mL hexane were mixed under sonication for 2 h and dried in flowing N_2_. The carbon‐loaded nanoparticles were heated at 5 °C min^−1^ to 200 °C (for partially spreading the islands) or 400 °C (for fully spread to obtain Pt‐string) under flowing compressed air, and maintained for 2 h to remove any residual organic surfactants, before being switched to flowing 5% H_2_/N_2_ and maintained at the annealed temperature for another 2 h.

### Structural Characterization

Low‐resolution scanning/transmission electron microscopy (S/TEM) imaging was performed on a JEOL JEM‐F200 operated at 200 kV. High‐resolution STEM imaging and energy‐dispersive X‐ray spectroscopy (EDX) mapping were performed on a double‐aberration corrected JEOL JEM‐ARM300F2 operated at 60 kV. The TEM was equipped with dual JEOL EDX detectors with 1.5 sr solid angle. High‐angle annular dark‐field (HAADF) STEM images were recorded at inner collection angle of ≈75 mrad. Atomic‐resolution TEM images were acquired at 300 kV operation on the same JEM‐ARM300F2, using a Gatan K3 camera for image acquisition under low‐dose conditions. TEM specimens of Pt‐island on Ru nanoparticles were prepared by drop‐casting a suspension of nanoparticles in toluene onto carbon‐coated copper grids or graphene layer supported on holey silicon nitride membrane on windowed silicon discs. The grids/discs were annealed following the conditions in the synthesis procedure to prepare partially spread Pt‐island and Pt‐string on Ru TEM specimens. Statistical analysis of particle dimension and distribution was performed on at least 100 Ru nanoparticles or 100 Pt islands using ImageJ.^[^ ^13^ ^]^

X‐ray absorption spectroscopy (XAS) measurements were performed at the XAS beamline at the National Synchrotron Light Source II, 7‐BM QAS beamline. The carbon‐loaded nanoparticles were measured using transmission geometry and probed from ≈150 eV below to ≈800 eV above the edge. Pt foil, Ru foil, and Pt‐island on Ru sample were measured in air under ambient conditions, while portions of Pt‐island on Ru sample were annealed and measured under 5% H_2_/N_2_ mixture at 200 and 400 °C, for measurements of the partially spread Pt‐island and Pt‐string on Ru samples, respectively. Data was processed and modelled using the Demeter XAS software package (Athena and Artemis).^[^ ^14^ ^]^ For EXAFS modelling, fcc structure corresponding to bulk Pt was used to generate Pt‐Pt and Pt‐Ru paths by replacing one Pt atom neighboring the X‐ray absorbing Pt with Ru atom. The amplitude reduction factor ($S_{0}^{2}$) for Pt (0.83) was obtained by EXAFS data analysis of Pt foil and subsequently fixed in the fits of the Pt edge data of different samples.^[^ ^15^ ^]^

X‐ray photoelectron spectroscopy (XPS) was performed on a Thermo Scientific ESCALAB250Xi featuring a monochromatic Al Kα source (1486.6 eV) operated at 120 W (13.8 kV × 8.7 mA) with a spot size of 500 µm and take‐off angle of 90°. The pass energy was 100 eV for the survey scans and 20 eV for the region scans. Data was processed and analyzed using CasaXPS software (V2.3.25).^[^ ^16^ ^]^ The peaks were calibrated to C 1s peak at 284.6 eV.^[^ ^17^ ^]^ Curve fitting was carried out with a mixture of Gaussian–Lorentzian functions and asymmetric line shapes for metallic peaks.

### Electrochemical Studies

Catalyst ink of the carbon‐supported Pt on Ru nanoparticles was prepared by dispersing 1 mg carbon‐loaded nanoparticles (10% metal loading) in a mixture of 280 µL Milli‐Q water, 100 µL isopropanol, and 20 µL Nafion, under sonication for 2 h to obtain a homogeneous suspension. Electrochemical measurements were conducted using a three‐electrode system with graphite rod as counter and Hg|HgO|KOH (1.0 м) as reference electrodes. The working electrode was prepared by drop‐casting 15 µL ink onto a glassy carbon rotating disk electrode (RDE, geometric surface area = 0.196 cm^2^, Pine Research Instrumentation) to give a Pt mass loading of 0.8 µg cm^−2^ and a Pt + Ru mass of 120 µg cm^−2^ on the electrode, which was then dried at 65 °C for 10 min. Measurements were performed using a µAutolab potentiostat controlled with Nova 2.1.2 software. During measurements, the working electrode was maintained at 1600 rpm in the electrolyte to remove any generated gases. The 1.0 м KOH electrolyte was saturated with N_2_ bubbled for 30 min prior to measurements. Catalyst surface activation was performed by cyclic voltammetry (CV) between −0.5 and −1.3 V (vs Hg|HgO) at a scan rate of 50 mV s^−1^ for five cycles before activity measurement. The HER activity was measured with potentials scanned from −0.5 to −1.3 V (vs Hg|HgO) in the cathodic direction at a scan rate of 10 mV s^−1^. All potentials were converted to standard reversible hydrogen electrode (vs RHE) potentials and currents were 95% iR compensated and capacitance corrected with the uncompensated electrolyte resistance determined by electrochemical impedance spectroscopy.

Mass activity per Pt was determined by normalizing the current at 70 mV (vs RHE) to the loaded mass of Pt on the electrode obtained from inductively coupled plasma mass spectrometry (ICP‐MS) on a PerkinElmer NexION 5000 spectrometer. To prepare for ICP‐MS measurement, the carbon‐supported nanoparticle catalyst was digested in aqua regia at 80 °C for 2 h. For the mass activity per Pt + Ru, due to the incomplete solubility of Ru even in strong acid and base, mass of Ru was determined from the ratio of Pt:Ru obtained from EDX spectral analysis, with the mass of Pt obtained from ICP‐MS.

The turnover frequency (TOF) values were calculated using the following equation^[^ ^18^ ^]^:

(1) $$\mathrm{TOF}=\frac{j}{n\;\times F\;\times \;x}$$

where *j* is the current density at the overpotential for HER to be evaluated, *n* is the number of electrons transferred to generate one mole of product (for HER, *n* = 2 for 1 mol H_2_ production), *F* is the Faraday constant (96500 C mol^−1^), *x* is the number of moles of active sites available for catalysis, which was deduced from the integration of CO oxidation peak and using the conversion factor of 420 µC cm^−2^.

CO stripping was conducted by applying 0.3 V (vs RHE) for 5 min in CO‐saturated 0.1 м HClO_4_, followed by three cycles of CV between 0.0 and 1.2 V (vs RHE) at 50 mVs^−1^. Stability test was evaluated by chronopotentiometry measured at a geometric current density of 10 mA cm^−2^ in 1.0 м KOH with other experimental conditions kept the same.

### Computational Details

Spin‐polarized density functional theory (DFT) calculations were performed using the Vienna ab initio simulation package (VASP),^[^ ^19^ ^]^ with the Perdew–Burke–Ernzerhof (PBE) functional^[^ ^20^ ^]^ within the generalized gradient approximation (GGA). In addition, the description of van der Waals (vdW) interactions were improved by using the D3BJ dispersion correction method.^[^ ^21^ ^]^ The projector‐augmented‐wave (PAW) method^[^ ^22^ ^]^ was used to describe the ionic cores while a plane wave basis set with a cutoff energy of 400 eV was used to model the valence electrons.

The energy of formation (*E_form_*) was calculated using the equation:

(2) $$E_{form}=E_{Ru\_xPt}-E_{Ru}-\left(\frac{E_{Pt\left(bulk\right)}}{n}\right)\times x$$

where *E_Ru_xPt_* is the energy of Pt configuration on the Ru(0001) surface with *x* number of Pt atoms, *E_Ru_* is the energy of Ru surface, *E_Pt(bulk)_* represents the energy of bulk Pt, and *n* is the number of Pt atoms in the bulk unit cell.

The Pt configuration investigations were performed with Pt atoms adsorbed on the hcp sites of the Ru surface (Figure S28, Supporting Information). Further explanation to the calculation is provided in the Experimental Section (Supporting Information) on DFT Calculations.

The Gibbs free energies in alkaline conditions were calculated using the equation:

(3) $$\Delta G=\Delta E_{DFT}+\Delta E_{ZPE}\;-\;T\Delta S$$

where Δ*E_DFT_* is the electronic energy difference of the respective systems, Δ*E_ZPE_* and Δ*S* refer to the difference in zero‐point energies and entropies at 298.15 K, respectively. The zero‐point energy and entropy values for gaseous molecules were taken from the NIST database.^[^ ^23^ ^]^ Herein, the Volmer–Tafel mechanism was adopted to describe HER in the alkaline media for the proposed configurations. The solvent effect was considered using the implicit solvation model as implemented within the VASPsol framework.^[^ ^24^ ^]^

## Conflict of Interest

The authors declare no conflict of interest.

## Supporting information



Supporting Information

## Data Availability

The data that support the findings of this study are available from the corresponding author upon reasonable request.
