# Supplementary material for: How the Arrangement of Platinum Atoms on Ruthenium Nanoparticles Improves Hydrogen Evolution Activity
Source: Adv Mater. 2025 Jul 22;37(41):e09610. doi: 10.1002/adma.202509610 (PMC12531725; doi:10.1002/adma.202509610)
Supplement: Supplementary file 1 — Supporting Information [file ADMA-37-e09610-s001.pdf]

ADVANCED MATERIALS

Supporting Information

for *Adv. Mater.*, DOI 10.1002/adma.202509610

How the Arrangement of Platinum Atoms on Ruthenium Nanoparticles Improves Hydrogen Evolution Activity

Qinyu Li, Soshan Cheong, Agus R. Poerwoprajitno, Shuting Xiang, Anatoly I. Frenkel, Yuwei Yang, Nicholas M. Bedford, Sohaib Umer, Martina Lessio*, Ichiro Ohnishi, Zeno R. Ramadhan, Dale L. Huber, Liming Dai, Wolfgang Schuhmann, J. Justin Gooding* and Richard D. Tilley**

Supporting Information

How the Arrangement of Platinum Atoms on Ruthenium Nanoparticles Improves Hydrogen Evolution Activity

Qinyu Li,[1] Soshan Cheong,[2]* Agus R. Poerwoprajitno,[3] Shuting Xiang,[4] Anatoly I. Frenkel,[4,5] Yuwei Yang,[6] Nicholas M. Bedford,[6] Sohaib Umer,[1] Martina Lessio,[1]* Ichiro Ohnishi,[7] Zeno R. Ramadhan,[2] Dale L. Huber,[3] Liming Dai,[6] Wolfgang Schuhmann,[8] J. Justin Gooding[1,9]* and Richard D. Tilley[1,2]*

[1]School of Chemistry, University of New South Wales, Sydney, NSW, 2052, Australia
[2]Mark Wainwright Analytical Centre, University of New South Wales, Sydney, NSW, 2052, Australia
[3]Center for Integrated Nanotechnologies, Sandia National Laboratories, Albuquerque, NM 87185, USA
[4]Department of Materials Science and Chemical Engineering, Stony Brook University, Stony Brook, NY, 11794, USA
[5]Division of Chemistry, Brookhaven National Laboratory, Upton, NY, 11973, USA
[6]School of Chemical Engineering, University of New South Wales, Sydney, NSW, 2052, Australia
[7]JEOL Ltd., 3-1-2 Musashino, Akishima, Tokyo, 196-8558, Japan
[8]Analytical Chemistry – Center for Electrochemical Sciences (CES), Faculty of Chemistry and Biochemistry, Ruhr University Bochum, D-44780, Bochum, Germany
[9]Australian Centre for NanoMedicine, University of New South Wales, Sydney, NSW, 2052, Australia
*To whom correspondence should be addressed. Soshan Cheong (s.cheong@unsw.edu.au); Martina Lessio (martina.lessio@unsw.edu.au); J. Justin Gooding (justin.gooding@unsw.edu.au); Richard D. Tilley (r.tilley@unsw.edu.au)

This Supporting Information file includes:

Supporting Experimental Section
Supporting Figures 1-28
Supporting Tables 1-4
Supporting Reference

Supporting Experimental Section

Supporting Experimental Section on DFT Calculations:
The energy of formation (E_{form}) refers to the energy required for the addition of one Pt atoms to an existing Pt configuration on the Ru(0001) surface. We first determined the most favorable site for a Pt atom adsorption on the Ru(0001) surface among three possible surface sites, hcp, fcc, and Ru-top site (Figure S28). The E_{form} values suggest that the hcp site is the most stable adsorption site for a single Pt atom, consistent with previous literature findings.[1] Therefore, investigations on the formation of further Pt configurations were focused on the hcp sites. We observed that the E_{form} value for a single Pt atom on the Ru(0001) surface is positive, indicating that adsorption is not favored (compared to bulk Pt).
The Ru(0001) was chosen as a representative surface because the Ru hourglass substructures are predominantly composed of low-index facets, such as Ru(0001). The Ru(0001) surface was modelled using a 6 × 6 supercell, which consists of three layers of Ru metal and a vacuum space of 15 Å in the z-direction to avoid interaction between the periodic images of the slab. In all calculations the adsorbate and the top two layers of Ru were allowed to relax. Dipole interactions were applied in the direction perpendicular to the slab. The surface Brillouin-zone integrations were approximated by sampling k-points according to the Γ-centered Methfessel-Paxton grid with size of 3 × 3 × 1. For structural optimization, the force convergence criterion was set to 0.03 eV $Å^{-1}$.

Supporting Figures

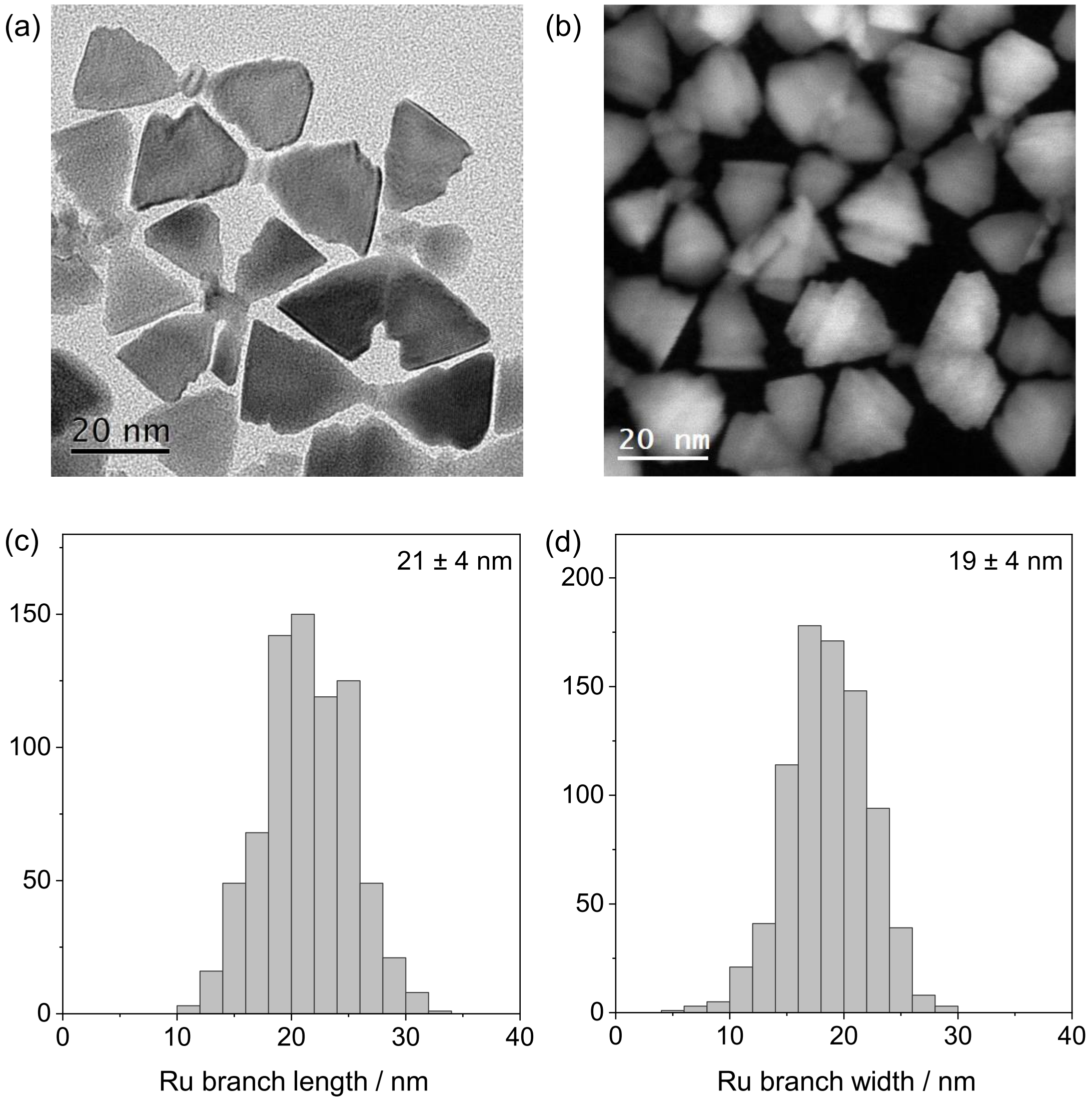

Figure S1. a) TEM and b) STEM images of hourglass Ru nanoparticles. Histogram of the c) length and d) width of Ru branches on the hourglass nanoparticles.

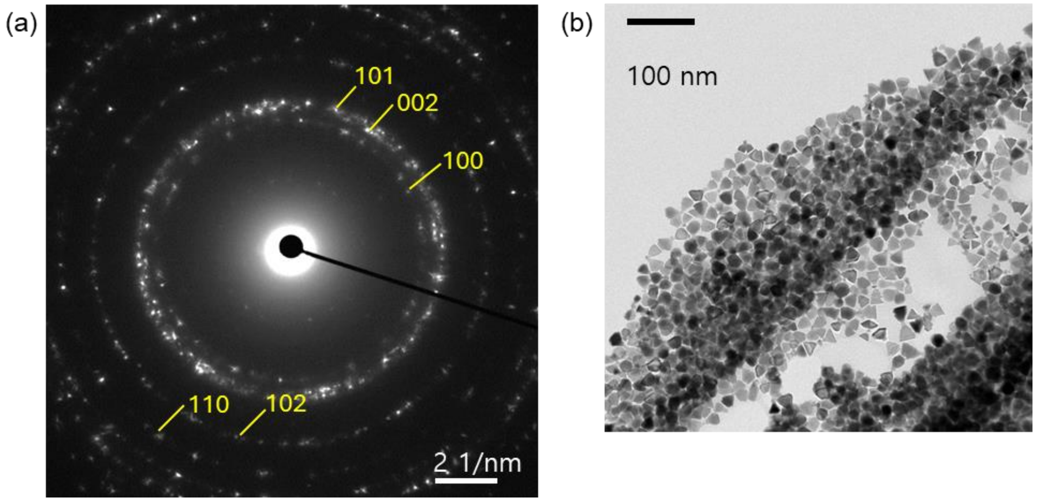

Figure S2. a) SAED pattern of an ensemble of hourglass nanoparticles indexed to hcp Ru. b) TEM image showing the area where the SAED pattern was acquired.

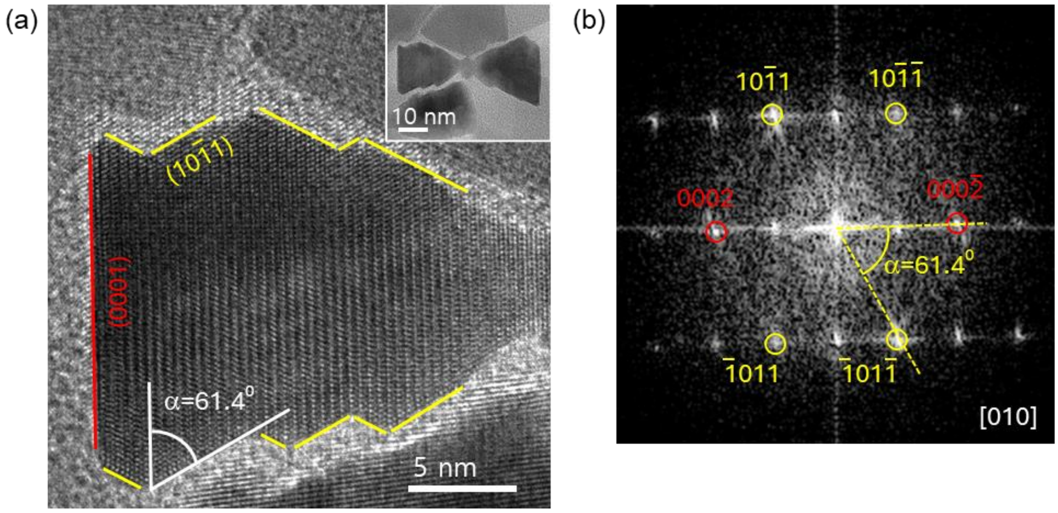

Figure S3. a) HRTEM image and b) the corresponding FFT of a branch on an hourglass Ru nanoparticle (inset in a). The FFT can be indexed to the diffraction spots of a hcp single crystal oriented on the [010] zone axis, indicating that the branch is bound predominantly by $\{0001\}$ and $\{10\bar{1}1\}$ facets.

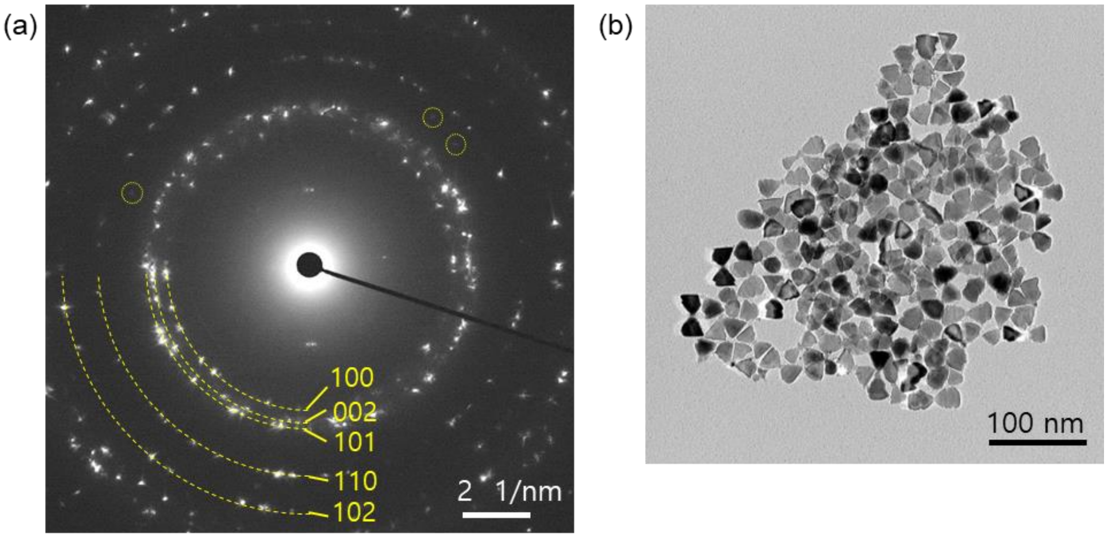

Figure S4. a) SAED pattern of an ensemble of Pt islands on Ru nanoparticles indexed to hcp Ru. The yellow circles mark the diffraction spots measured to be 5.26 nm^{-1}, which corresponds to the (200) of fcc Pt. The small crystallite size of the Pt islands is indicated by the low Pt diffraction signals and only Pt(200) spots are discernible, as the other Pt d-spacings are closely spaced with those of Ru. b) TEM image showing the area where the SAED pattern was acquired.

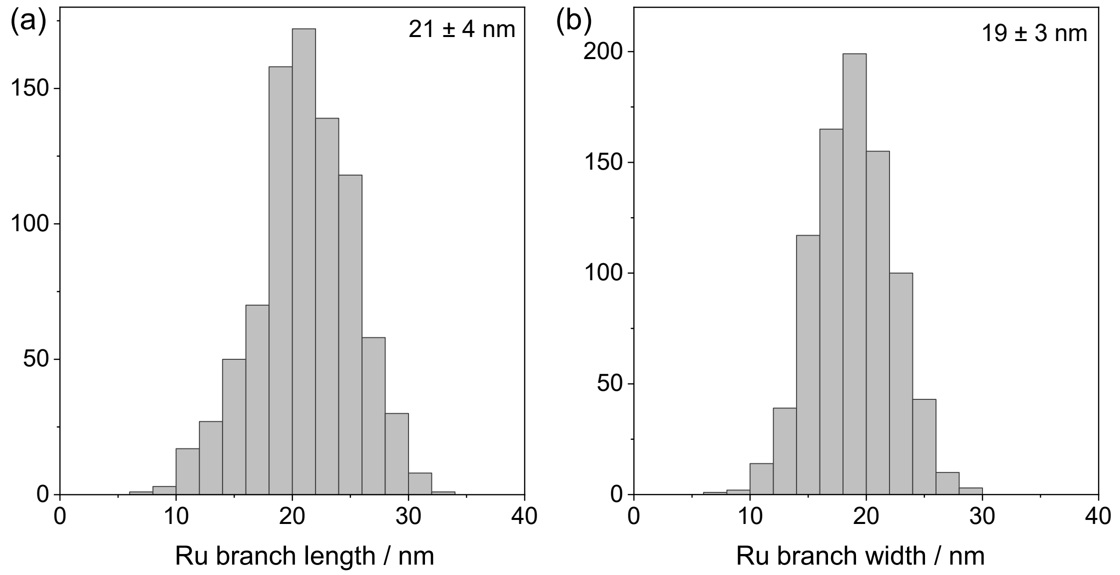

Figure S5. Histogram of the a) length and b) width of Ru branches measured on Pt-island on Ru nanoparticles.

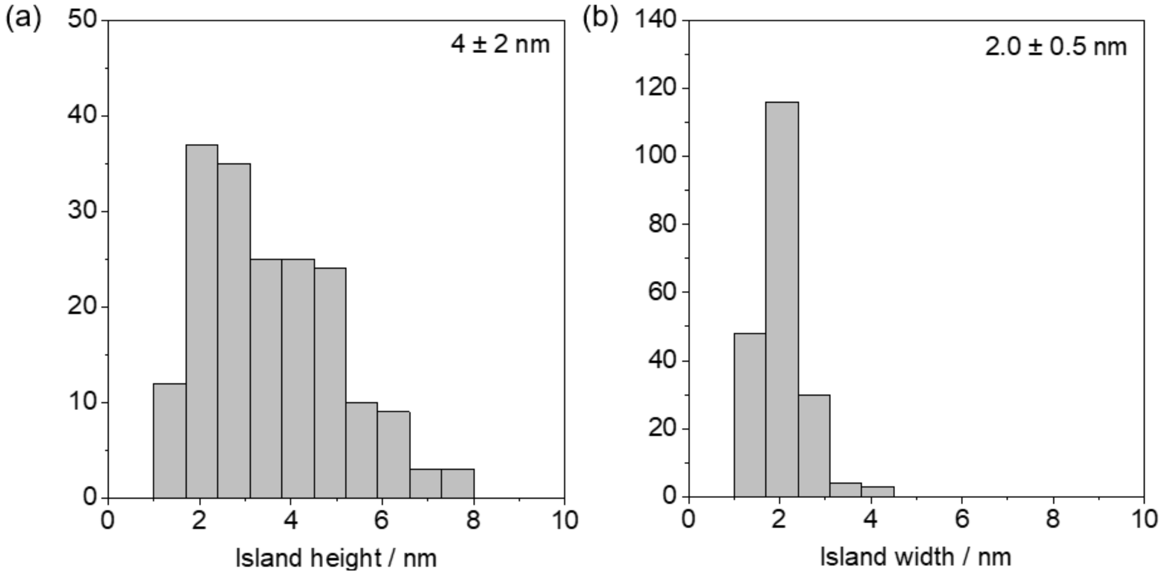

Figure S6. Histogram of the a) height and b) width of Pt islands on Ru nanoparticles.

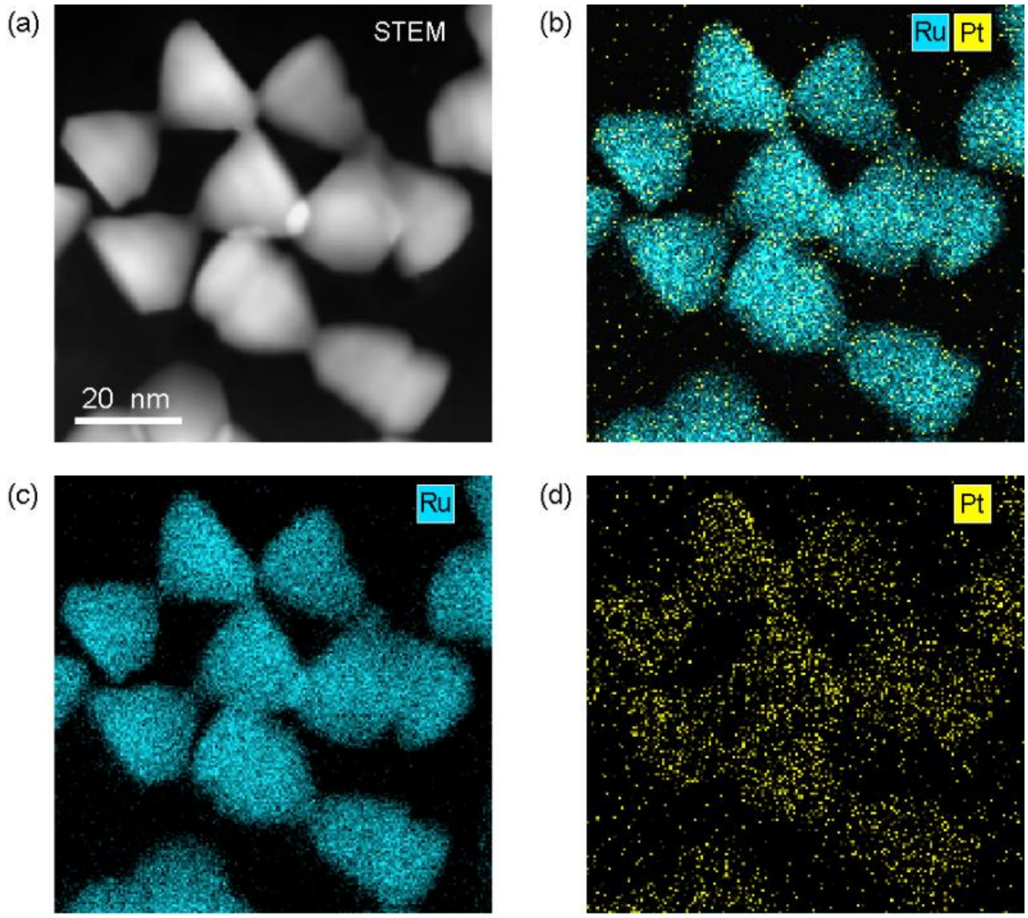

Figure S7. a) STEM image, b) EDX overlay map and individual elemental c) Ru and d) Pt maps of a group of Pt-string on Ru nanoparticles.

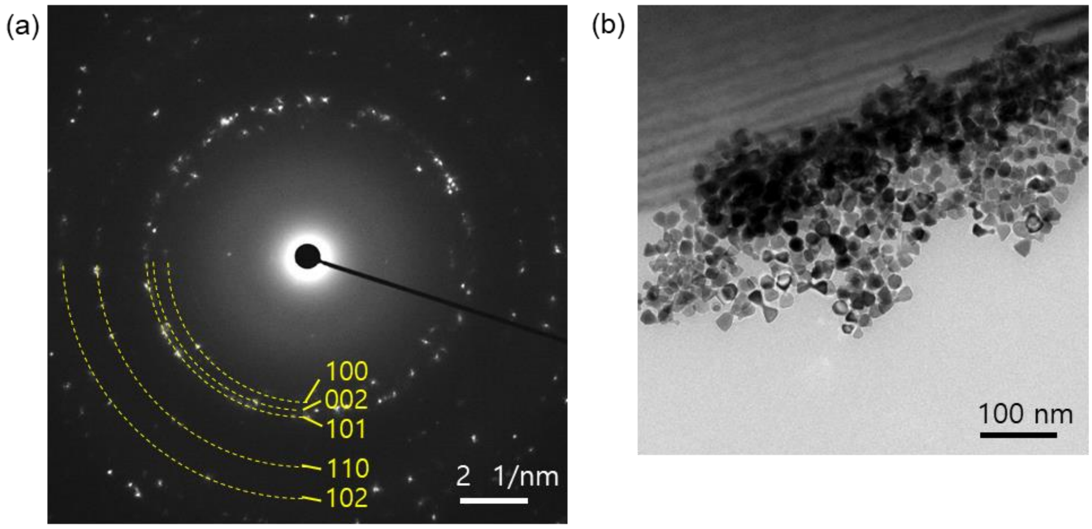

Figure S8. a) SAED pattern of an ensemble of Pt-decorated Ru nanoparticles after annealing at 400 °C. The pattern can be indexed to hcp Ru. b) TEM image showing the area where the SAED pattern was acquired.

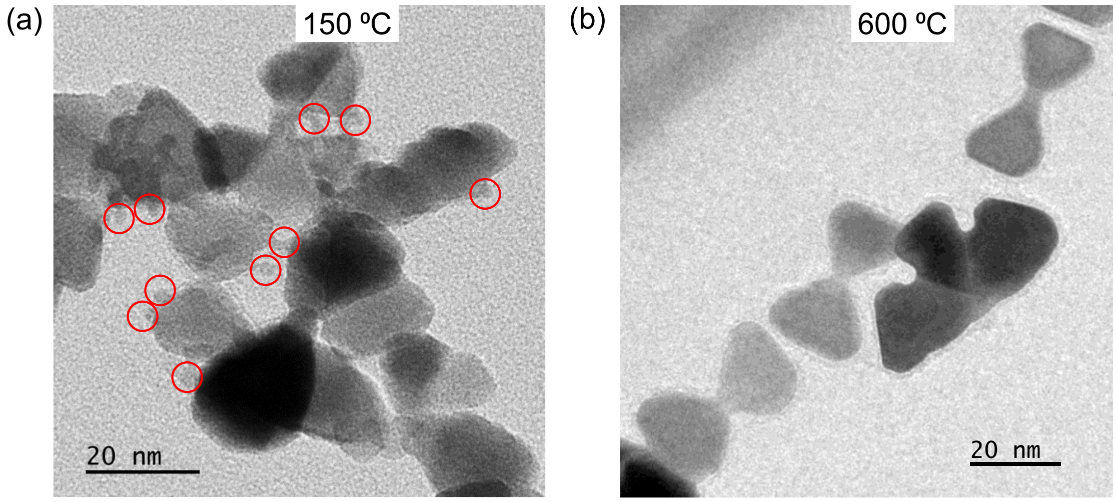

Figure S9. TEM images of Pt-decorated Ru nanoparticles after annealing in a 5%H_2/N_2 atmosphere at a) 150 °C and b) 600 °C. The red circles in (a) mark the Pt islands on the Ru nanoparticles.

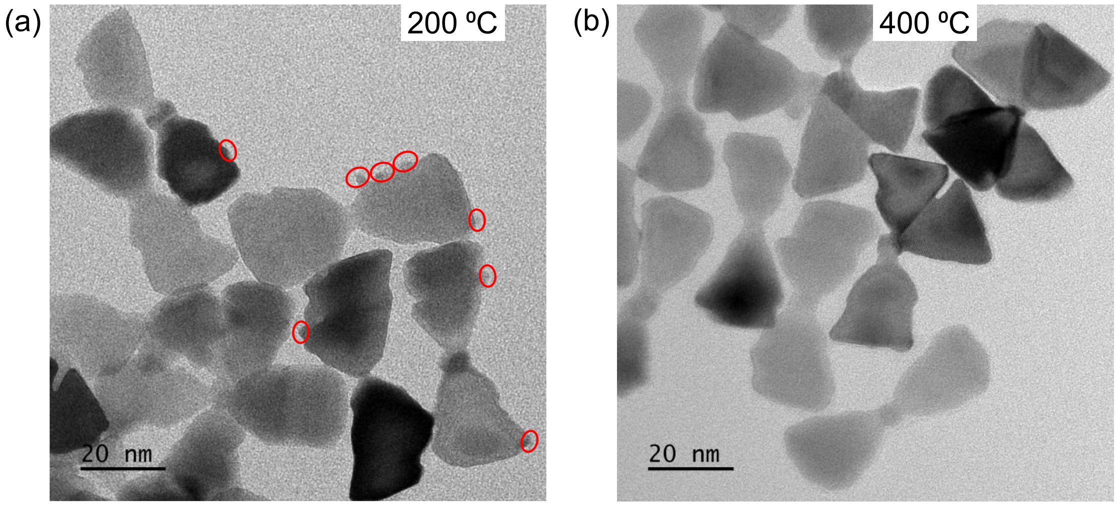

Figure S10. TEM images of Pt-decorated Ru nanoparticles after annealing in a 5% H_2/N_2 atmosphere a) at 200 °C for 10 hours, where some partially spread Pt islands can still be observed (marked by red ovals), and b) at 400 °C for 5 hours.

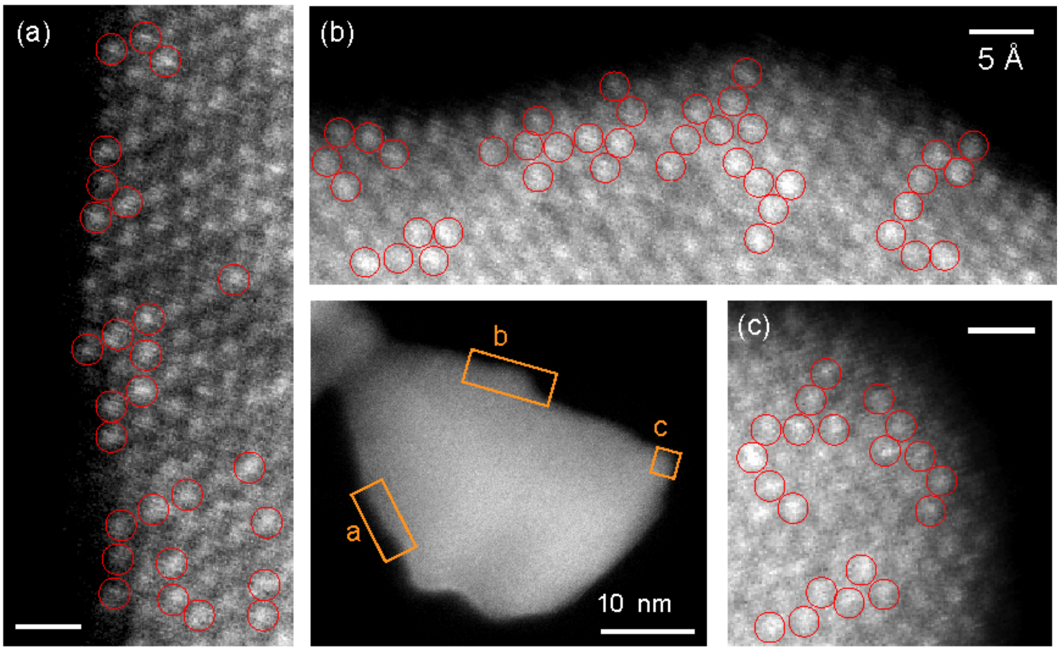

Figure S11. High-resolution HAADF-STEM images acquired near the edges of a Ru hourglass branch after annealing at 400 °C, showing brighter contrast atoms in string and cluster arrangements, as marked by the red circles. Some single atoms and dimers are also observed. Orange boxes a-c indicates areas where the respective high-resolution STEM images in panels (a-c) were taken.

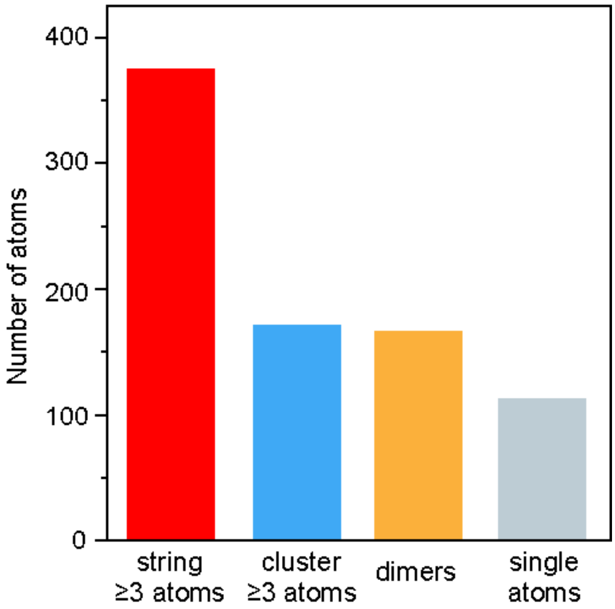

Figure S12. Distribution of Pt atom configurations obtained from HAADF-STEM image analysis.

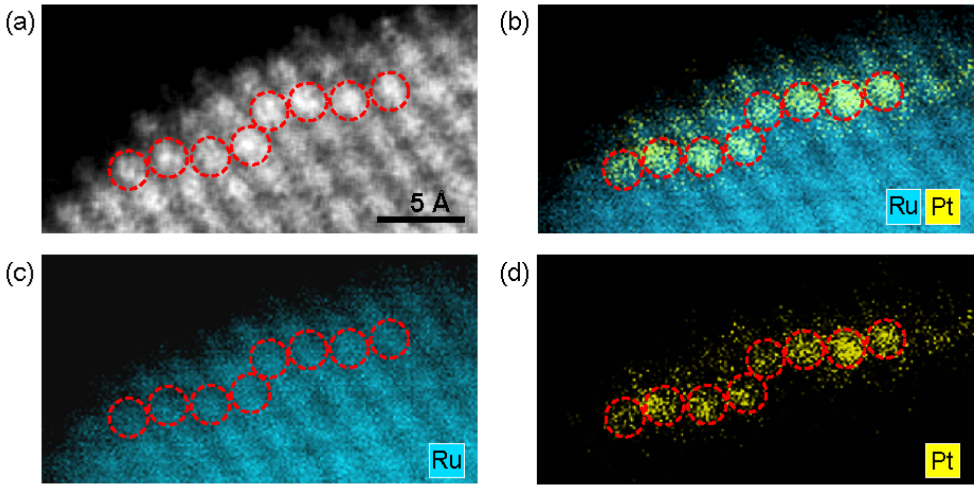

Figure S13. High-resolution STEM-EDX analysis: a) HAADF-STEM image, b) overlay map and elemental c) Ru and d) Pt maps of the Pt-string on Ru nanoparticle shown in Figure 2a. The red circles mark the position of Pt atoms as identified by the brighter atoms in (a) and localization of Pt EDX signal in (d).

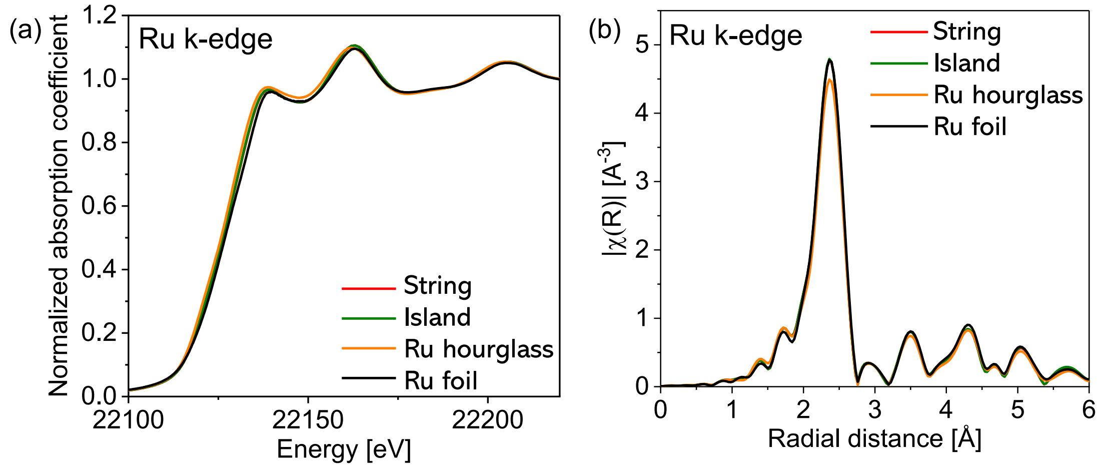

Figure S14. a) Normalized XANES spectra and b) Fourier transform EXAFS spectra of Ru foil, Ru hourglass, Pt-string on Ru and Pt-island on Ru samples at the Ru K-edge.

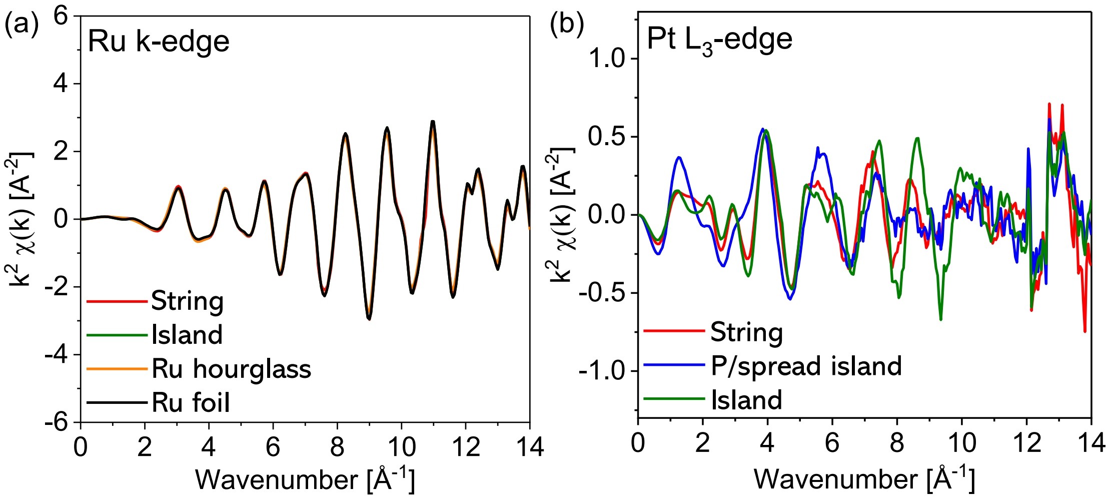

Figure S15. a) k^2-weighted EXAFS spectra in k space for Ru of Ru foil, Ru hourglass, Pt-string on Ru and Pt-island on Ru samples. b) k^2-weighted EXAFS spectra in k space for Pt of Pt-string, partially spread Pt-island and Pt-island on Ru samples.

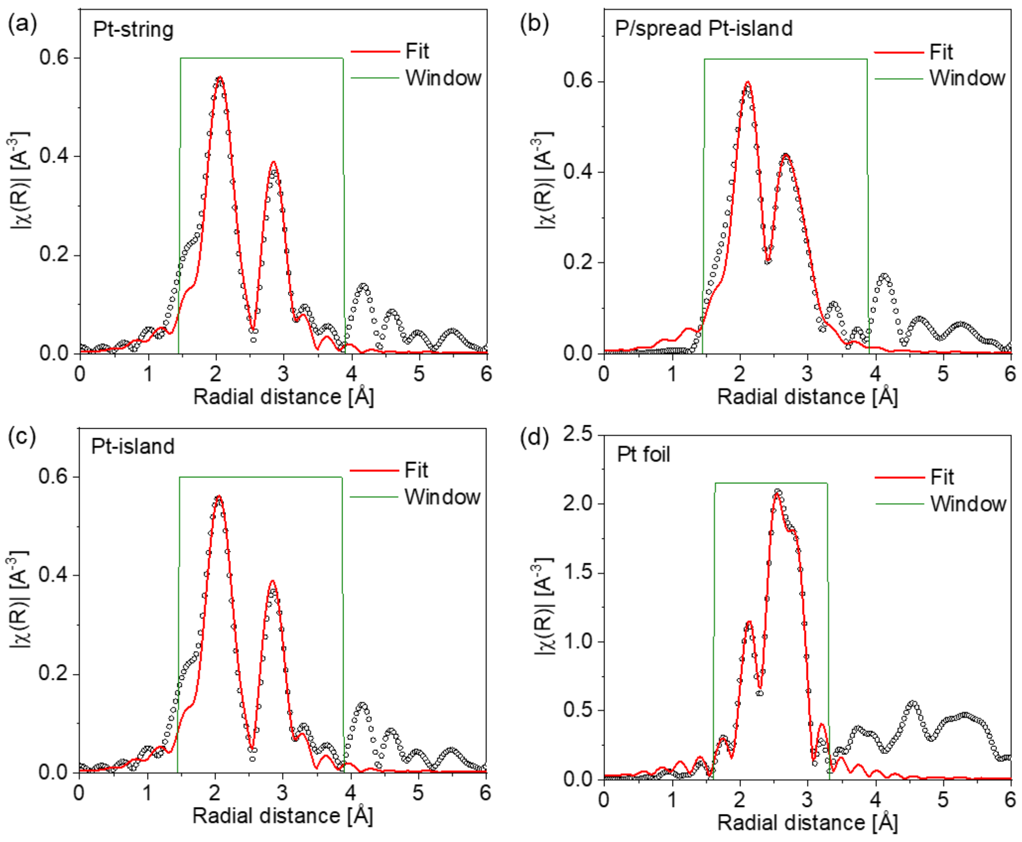

Figure S16. Fitted R-space spectra for Pt L_3-edge of a) Pt-string, b) partially spread Pt-island and c) Pt-island on Ru samples and d) Pt foil. Pt-Ru and Pt-Pt paths were used in the fitting for (a), (b) and (c), as shown in Figure S17 below. For (d) only Pt-Pt path was used.

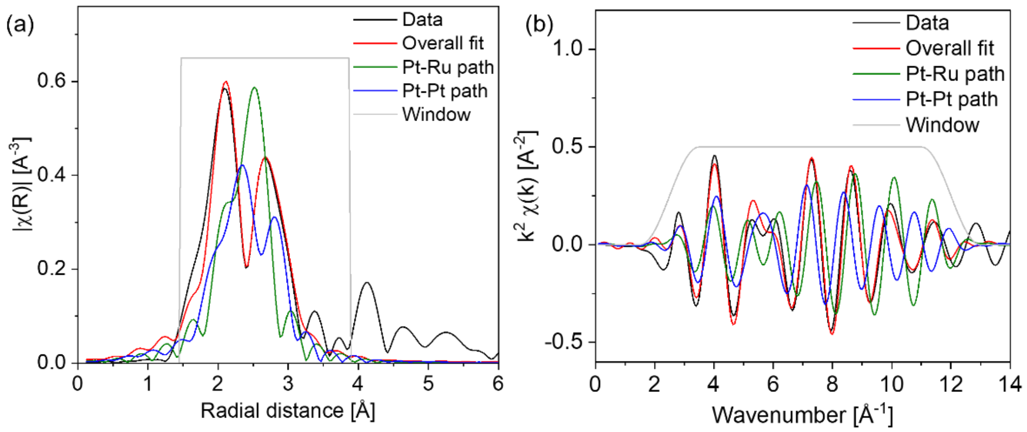

Figure S17. a) Fitted R-space spectra for Pt L_3-edge of partially spread Pt-island on Ru sample, showing partial contributions of Pt-Ru and Pt-Pt paths, together with the overall theoretical fit. b) The corresponding fitted k^2 range for Pt L_3-edge plotted with the individual Pt-Ru and Pt-Pt contributions to the real part of the Fourier transform, demonstrating that these components correctly sum to the total signal.

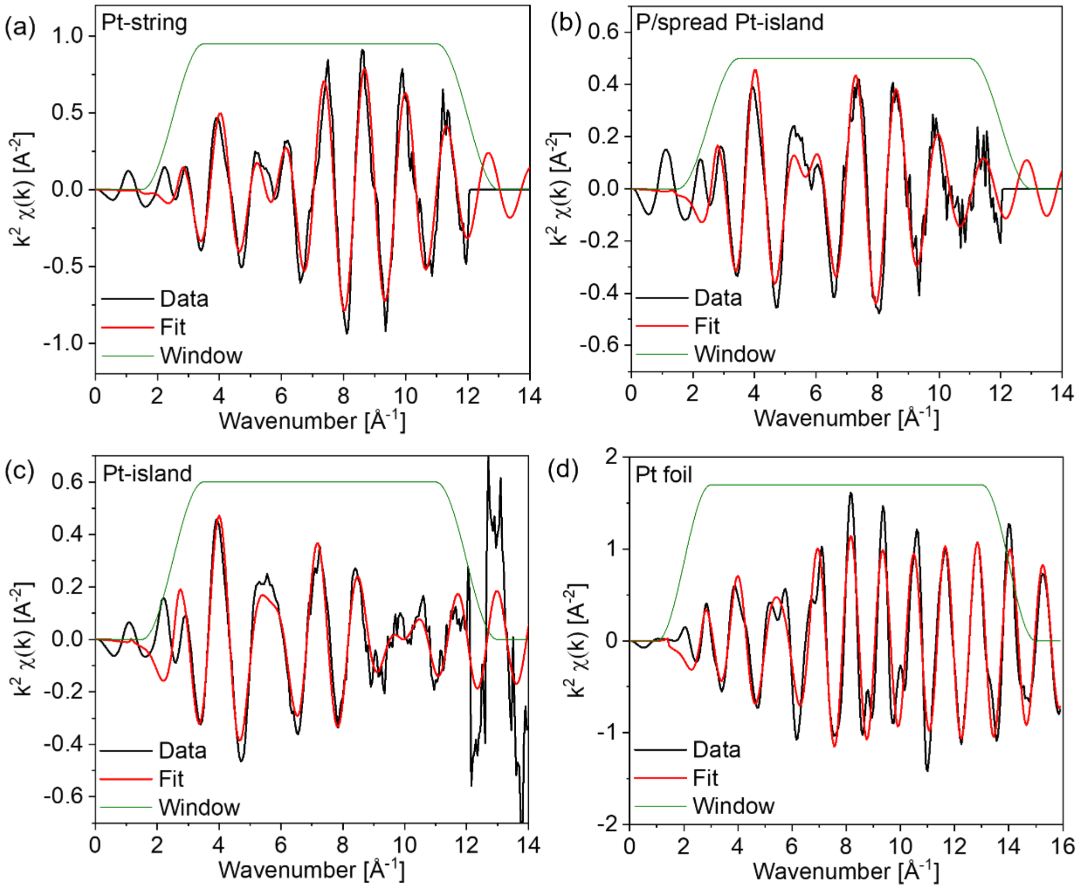

Figure S18. Fitted k^2 range for Pt L_3-edge of a) Pt-string, b) partially spread Pt-island and c) Pt-island on Ru samples, and d) Pt foil.

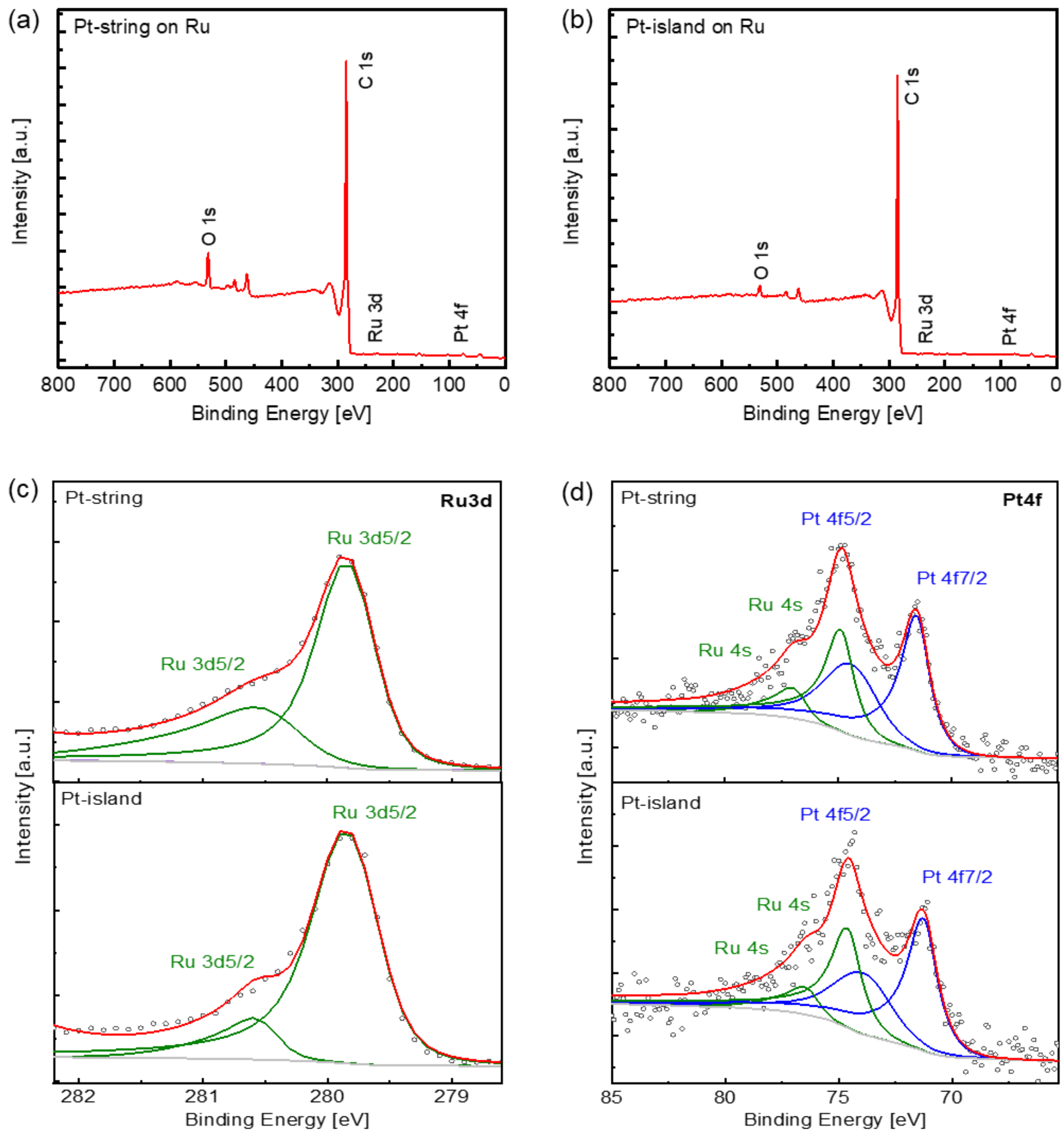

Figure S19. XPS analysis: XPS survey scans of a) Pt-string on Ru and b) Pt-island on Ru nanoparticles, c) Ru 3d and d) Pt 4f and Ru 4s core level spectra for the Pt-string on Ru and Pt-island on Ru nanoparticles.

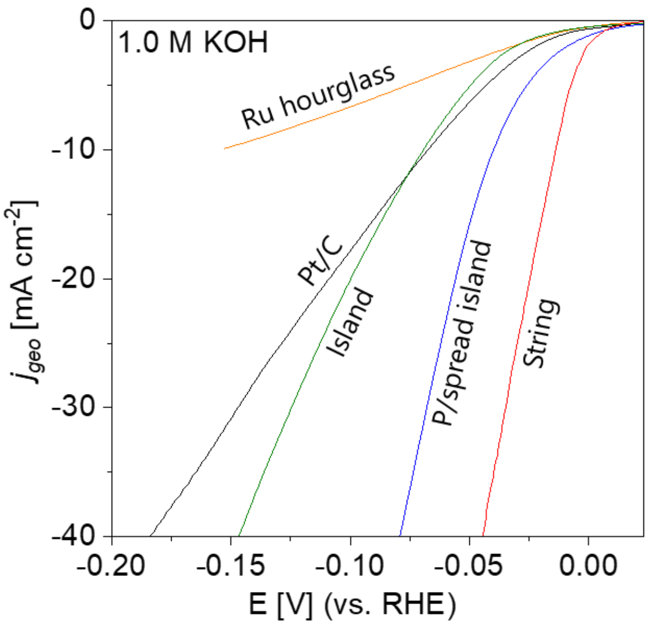

Figure S20. HER polarization curves showing activity comparison of carbon-supported Ru hourglass nanoparticles, commercial Pt/C, and carbon-supported Pt-island, partially spread Pt-island and Pt-string on Ru nanoparticle catalysts.

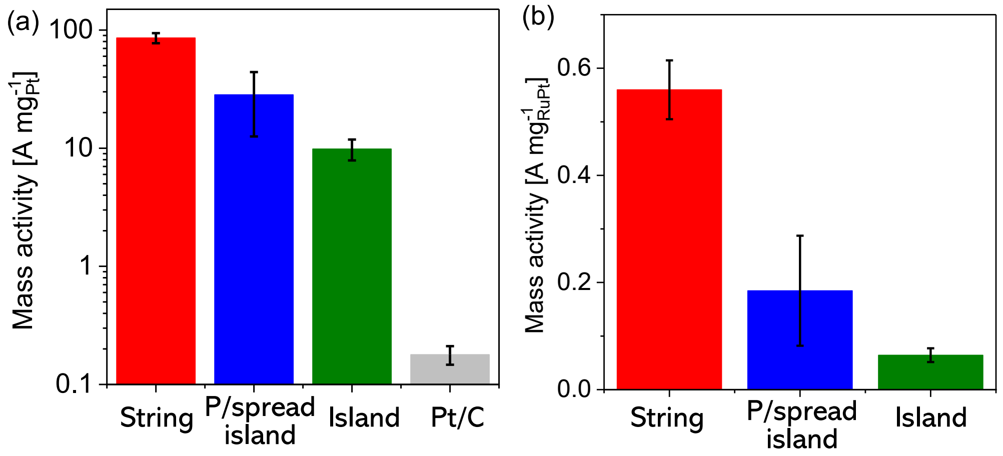

Figure S21. a) Mass activity of Pt-string, partially spread Pt-island and Pt-island on Ru, and commercial Pt/C catalysts measured at overpotential of 70 mV, normalized by the mass of Pt. b) Mass activity of Pt-string, partially spread Pt-island and Pt-island on Ru catalysts measured at the overpotential of 70 mV, normalized by the total mass of Pt and Ru.

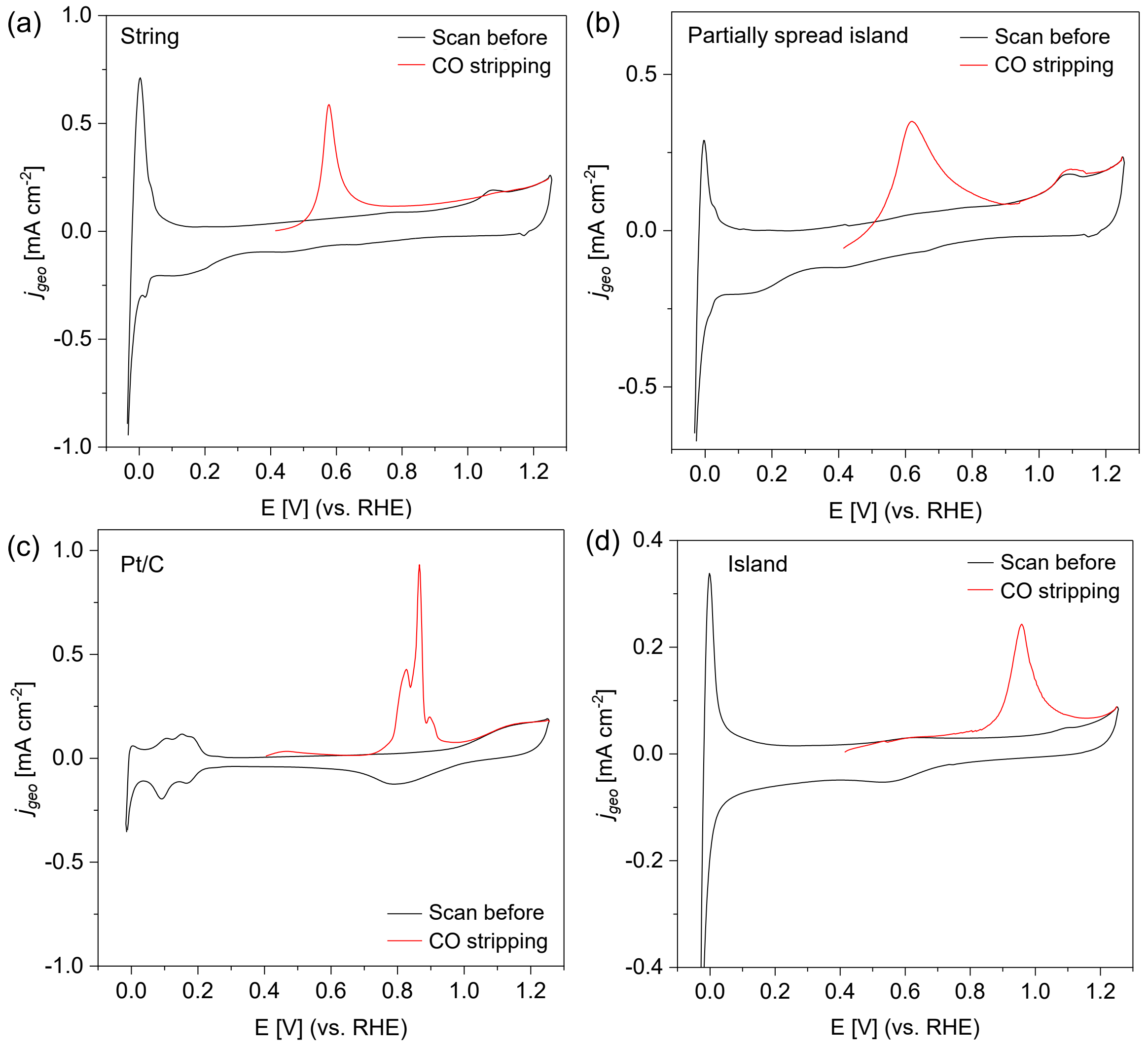

Figure S22. CO stripping-voltammetry of a) Pt-string on Ru, b) partially spread Pt-island on Ru, c) commercial Pt/C, and d) Pt-island on Ru catalysts.

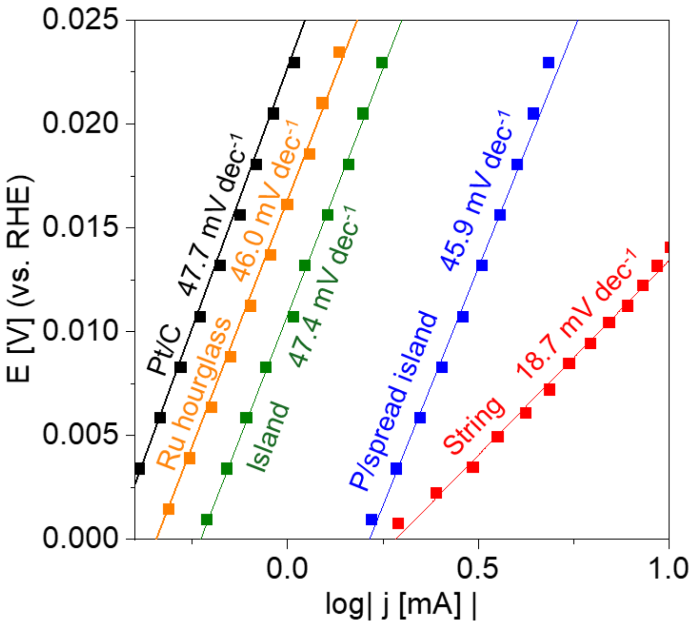

Figure S23. Tafel plots of Pt/C, Ru hourglass, Pt-island, partially spread Pt-island and Pt-string on Ru catalysts in 1.0 м KOH.

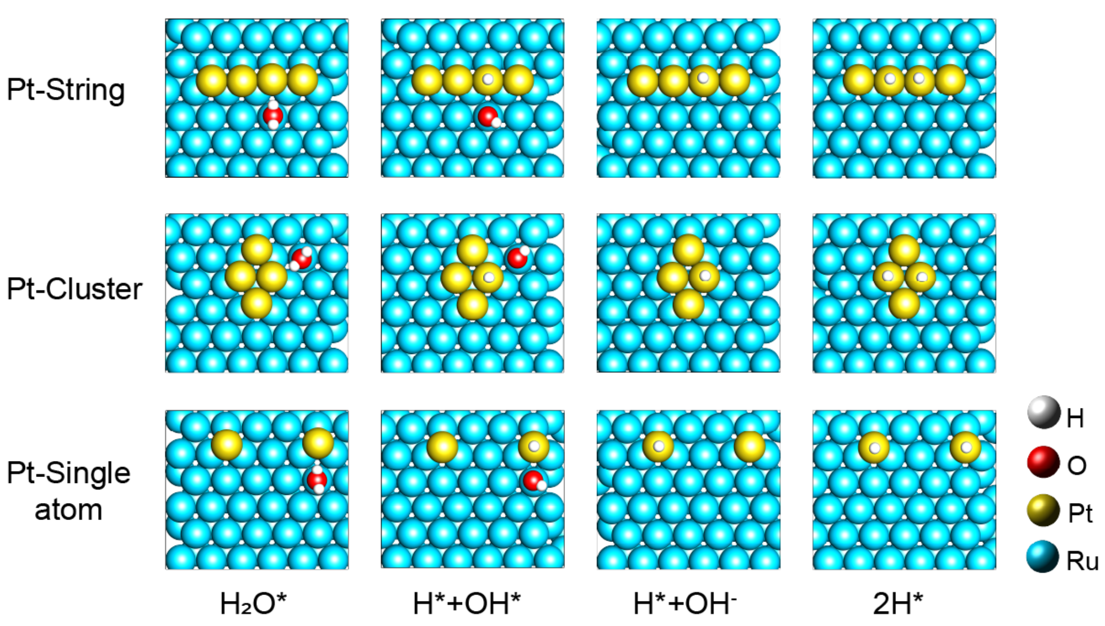

Figure S24. Optimized geometries of HER intermediates adsorbed on Pt-string, Pt-cluster, and Pt-single atom on a Ru(0001) surface.

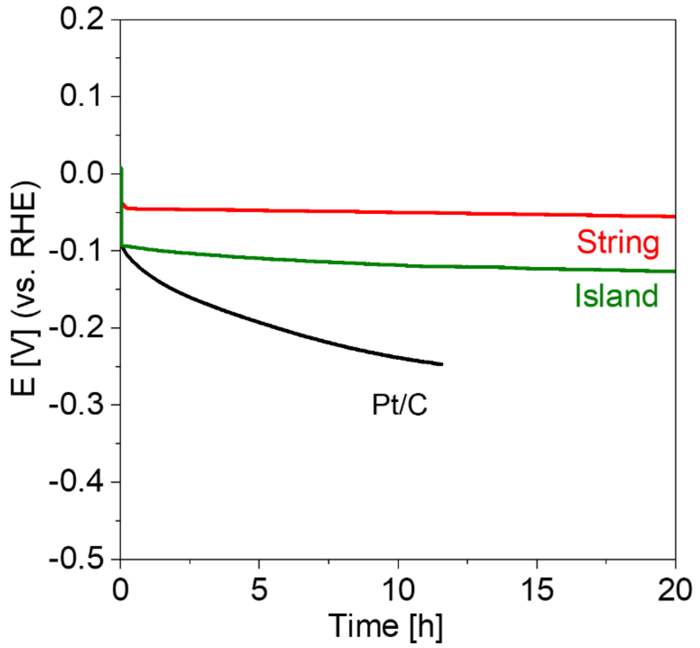

Figure S25. Chronopotentiometry of Pt-string on Ru and Pt-island on Ru catalysts compared to Pt/C at 10 mA cm^{-2} (vs. RHE) in 1 M KOH.

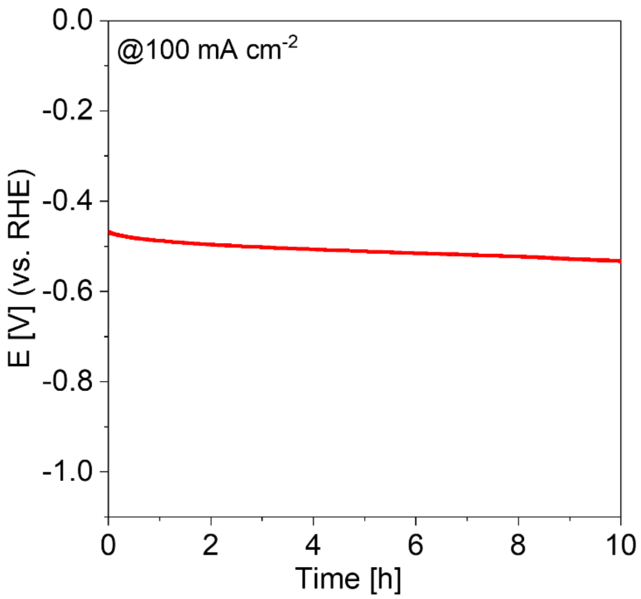

Figure S26. Chronopotentiometry plot of Pt-string on Ru catalyst at 100 mA cm^{-2} (vs. RHE) in 1 M KOH.

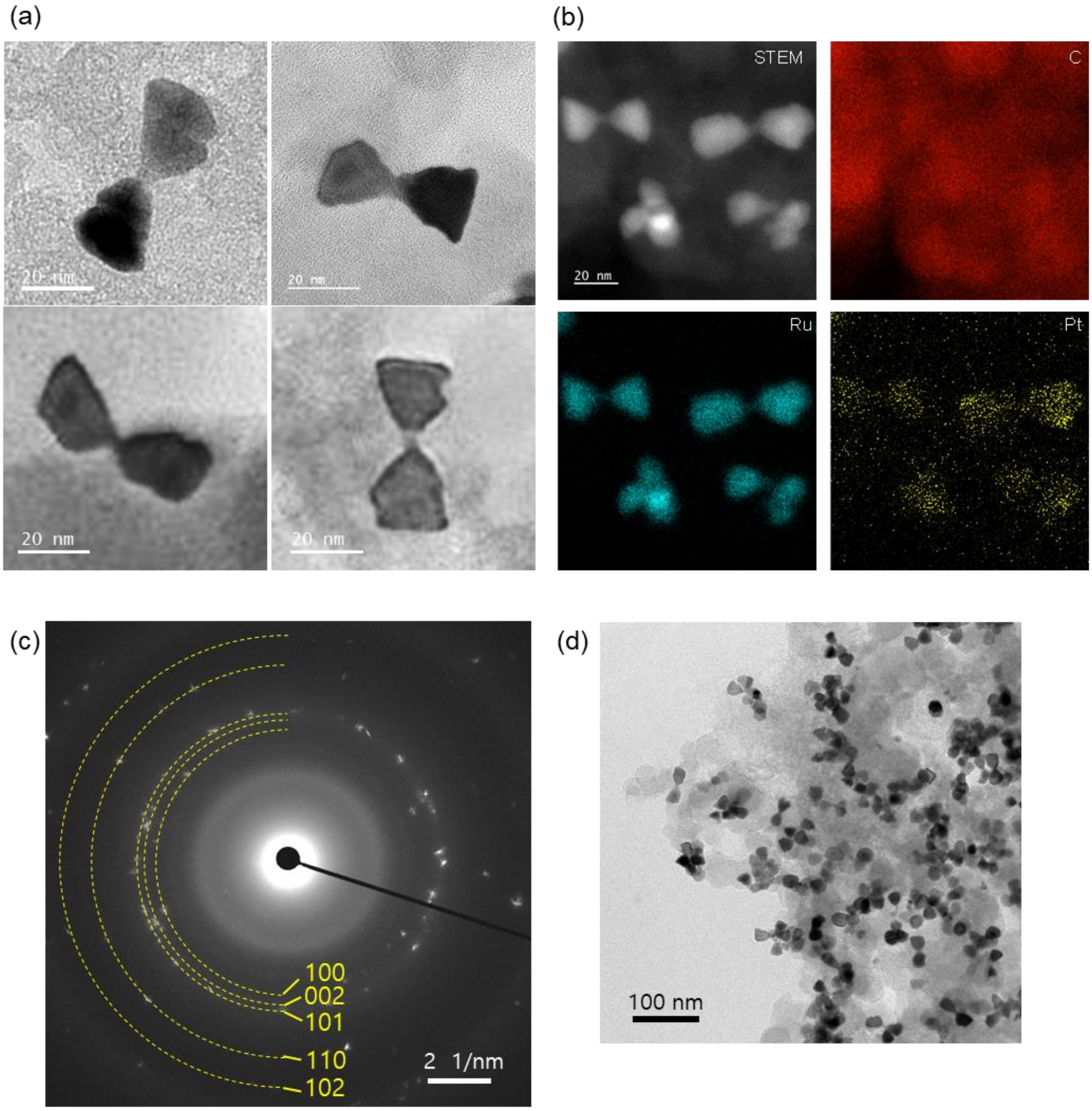

Figure S27. Post-catalysis characterization: a) TEM images, b) STEM-EDX elemental mapping analysis and c) SAED pattern of carbon-supported Pt-string on Ru nanoparticle catalyst after stability test at 10 mA cm^{-2} (vs. RHE) in 1 M KOH. d) TEM image of the area where the SAED pattern was acquired.

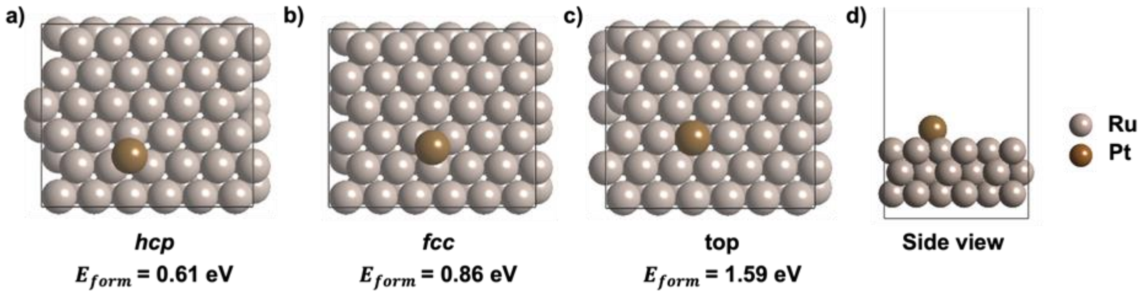

Figure S28. Adsorption geometry and the corresponding energy of formation (E_{form}) of a Pt atom at a) hcp, b) fcc and c) top sites on a Ru(0001) surface. d) Side view of a Pt atom at a hcp site on Ru(0001). E_{form} is defined according to Equation (1) in the main text.

Supporting Tables

Table S1. Measured Ru d-spacings (d_{hkl}) obtained from SAED patterns of Ru and Pt-decorated Ru nanoparticles.

	d_{hkl} [nm]		
hkl	Ru hourglass	Pt-island on Ru	Pt-island on Ru after 400 °C annealing
100	0.240	0.238	0.237
002	0.219	0.218	0.218
101	0.210	0.209	0.209
102	0.162	0.162	0.160
110	0.138	0.137	0.139

Table S2. DFT calculated Pt-Pt distances.

Pt on Ru(0001) configuration	Model	Energy of formation (E_{form}) [eV]	Pt-Pt distance, d(Pt-Pt) [Å]
Single atom	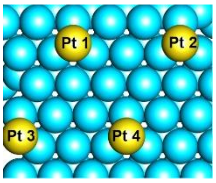	2.67	-
Dimer	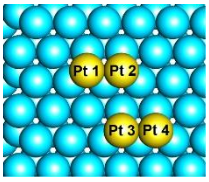	2.15	d(Pt1-Pt2) = 2.71<br>d(Pt3-Pt4) = 2.68
Cluster of 4	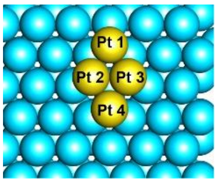	1.57	d(Pt1-Pt2) = d(Pt1=Pt3) = 2.77<br>d(Pt2-Pt4) = d(Pt3-Pt4) = 2.74
String of 4	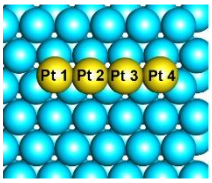	1.32	d(Pt1-Pt2) = d(Pt3-Pt4) = 2.66<br>d(Pt2-Pt3) = 2.64

Table S3. EXAFS fitting result for Pt L_3-edge.

	Contribution	Coordination number	R [Å]	σ^2 [Å^2]	ΔE [eV]
Pt-island on Ru	Pt-Ru	1.2(5)	2.66(2)	0.0042(22)	4.8(1.1)
	Pt-Pt	6.7(1.1)	2.70(1)	0.0094(16)	
Partially spread Pt-island on Ru	Pt-Ru	2.1(9)	2.67(1)	0.0042(24)	5.6(1.8)
	Pt-Pt	4.0(1.3)	2.68(2)	0.0055(24)	
Pt-string on Ru	Pt-Ru	3.6(1.0)	2.69(1)	0.0037(18)	7.5(1.7)
	Pt-Pt	2.3(1.2)	2.68(2)	0.0029(37)	

Table S4. HER activity comparison of recently reported Pt-Ru based catalysts and activity improvement with respect to commercial Pt/C in 1.0 M KOH electrolyte.

Catalyst	Overpotential at 10 mA cm^{-2} [mV]	Tafel slope[a] [mV dec^{-1}]	TOF[a] [H_2 s^{-1}]	TOF improvement	Reference
Pt-string on Ru	14	18.7	9.2 @70 mV<br>15.3 @100 mV	9.2	This work
Partially spread Pt-island on Ru	39	45.9	2.6 @70 mV	2.6	
Pt-island on Ru	69	47.4	1.7 @70 mV	1.7	
Pt/C	67	50.0	1.0 @70 mV		
RuPt nanoclusters ($Ru_{5.67}Pt/PC$)	12	39	18.58 @100 mV	8.8	*Chem. Commun.* **2024**, *60*, 7188. DOI: 10.1039/D4CC02382J
Pt/C	57	85	2.10 @100 mV		
PtRu on WO_3 ($PtRu/WO_3$-O_V)	9	25.4	1.33 @50 mV	5.7	*Adv. Energy Mater.* **2024**, *14*, 2402372. DOI: 10.1002/aenm.202402372
Pt/C	29	65.1	0.23 @50 mV		
hcp-RuPt alloy nanoparticles	12.9	23.9	2.35 @30 mV	5.3	*J. Am. Chem. Soc.* **2022**, *144*, 4224. DOI: 10.1021/jacs.2c00583
fcc-RuPt alloy nanoparticles	25.2	39.1	0.92 @30 mV	2.1	
Pt/C	42.1	67.5	0.44 @30 mV		
Pt-Ru alloy clusters (CNT-NPA-PtRu)	18.3	62.97	1.53 @100 mV	4.0	*Chem. Sci.* **2024**, *15*, 9851.DOI: 10.1039/D4SC00182F
Pt/C	33	NA	0.38 @100 mV		
PtRu clusters on acetylene black ($Pt_{0.47}$-Ru/Acet)	17	66.6	0.7 @100 mV	3.2	*Chem. Eng. J.* **2022**, *448*, 137611. DOI: 10.1016/j.cej.2022.137611
Pt/C	23	NA	0.22 @100 mV		
PtRu on defective carbon cloth (PtRu/CC-P)	44	45.1	2.60 @100 mV	2.5	*Nanoscale* **2022**, *14*, 15942. DOI: 10.1039/D2NR04369F
Pt/C	81	56.4	1.06 @100 mV		
N-doped Pt-Ru single atom alloy ((Ru–N)@Pt)	15	25	60.6 @100 mV	1.7	*J. Mater. Chem. A* **2021**, *9*, 14941. DOI: 10.1039/D1TA03593B
Pt/C	35	53	35.5 @100 mV		
Pt nanoclusters-Ru nanowires (Pt/Ru NWs)	24	26.3	1.42 @50 mV	1.22	*Adv. Powder Mater.* **2024**, *3*, 100214. DOI: 10.1016/j.apmate.2024.100214
Pt/C	33	45	1.16 @50 mV		

Catalyst	Overpotential at 10 mA cm^{-2} [mV]	Tafel slope[a] [mV dec^{-1}]	TOF[a] [H_2 s^{-1}]	TOF improvement	Reference
PtRu on carbon nanotubes (PtRu/mCNT)	15	33.5	NA	–	*Energy Environ. Sci.* **2022**, *15*, 102. DOI: 10.1039/D1EE02518J
Pt/C	39	38.3	NA		
Pt single atom on Ru/RuO_2	18	18.5	NA	–	*Nat. Commun.* **2024**, *15*, 1447. DOI: 10.1038/s41467-024-45654-9
Pt/C	45	50.7	NA		
Pt-Ru heterojunction cluster (PtRu/BNHCSs)	18	37.7	NA	–	*Adv. Energy Mater.* **2025**, *15*, 2405828. DOI: 10.1002/aenm.202405828
Pt/C	NA	37.9	NA		
PtRu on black phosphorus (PtRu NCs/BP)	22	19	NA	–	*ACS Catal.* **2019**, *9*. 10870. DOI: 10.1021/acscatal.9b03506
Pt/C	77	46	NA		

[a] NA = not available

Supporting Reference

[1] a) S. Sakong, J. M. Fischer, D. Mahlberg, R. J. Behm, A. Groß *Electrocatal.* **2017**, *8*, 530. b) M. Lischka, C. Mosch, A. Groß, *Electrochim. Acta* **2007**, *52*, 2219.
